# Anisotropic phenanthroline-based ruthenium polymers grafted on a titanium metal-organic framework for efficient photocatalytic hydrogen evolution

**DOI:** 10.1038/s42004-022-00763-8

**Published:** 2022-12-03

**Authors:** Spandana Gonuguntla, Saddam Sk, Anjana Tripathi, Ranjit Thapa, Gopinath Jonnalagadda, Chandrani Nayak, Dibyendu Bhattacharyya, S. N. Jha, Annadanam V. Sesha Sainath, Vijayanand Perupogu, Ujjwal Pal

**Affiliations:** 1 Department of Energy & Environmental Engineering, CSIR-Indian Institute of Chemical Technology, Hyderabad-, 500007 India; 2 Academy of Scientific and Innovative Research (AcSIR), Ghaziabad-, 201002 India; 3 Department of Physics, SRM University-AP, Amravati-, 522502 Andhra Pradesh India; 4 Polymers and Functional Materials, Fluoro-Agrochemicals Department, CSIR-Indian Institute of Chemical Technology, Hyderabad-, 500007 India; 5 Atomic and Molecular Physics Division, Bhabha Atomic Research Centre, Mumbai-, 400085 India; 6 Beamline Development and Application Section, Bhabha Atomic Research Centre, Mumbai-, 400085 India

**Keywords:** Photocatalysis, Energy harvesting, Porous materials, Metal-organic frameworks

## Abstract

Conjugated polymers and titanium-based metal-organic framework (Ti-MOF) photocatalysts have demonstrated promising features for visible-light-driven hydrogen production. We report herein a strategy of anisotropic phenanthroline-based ruthenium polymers (PPDARs) over Ti-MOF, a tunable platform for efficient visible-light-driven photocatalytic hydrogen evolution reaction (HER). Several analytical methods including X-ray absorption spectroscopy (XAS) revealed the judicious integration of the surface-active polymer over the Ti-MOF reinforcing the catalytic activity over the broad chemical space. PPDAR-4 polyacrylate achitecture led to a substantial increase in the H_2_ evolution rate of 2438 µmolg^−1^h^−1^ (AQY: 5.33%) compared to pristine Ti-MOF (238 µmol g^−1^ h^−1^). The separation of photogenerated charge carriers at the PPDAR-4/Ti-MOF interface was confirmed by the optical and electrochemical investigations. The experimental, as well as theoretical data, revealed their physical and chemical properties which are positively correlated with the H_2_ generation rate. This offers a new avenue in creating polymer-based MOF robust photocatalysts for sustainable energy.

## Abstract (web summary)

Combining conjugated polymers with transition-metal-based metal-organic frameworks offers an opportunity to produce efficient photocatalytic materials. Here, exposed active sites and efficient charge transfer lead to hydrogen evolution rates of up to 2438 µmol g^−1^ h^−1^ for composites of anisotropic phenanthroline-based ruthenium polymers grafted on titanium-based MOFs.

## Introduction

The development of various carbon-free technologies would highly favor the generation of sustainable energy^1^. Among the various classes of photocatalysts present so far, including organic and inorganic materials^2–4^, conjugated polymeric^5^, and MOF have emerged as prominent platforms recently. The fine-tuning of the electronic and structural properties of the polymers and the MOFs can be carried out through the synthesis. The development of polymeric and MOF networks, with enhanced porosity and suitable functional groups, and conjugated polymer moieties, pave different ways toward enhanced catalytic activity^6^. MOF is considered to be a class of porous materials with self-assembled conjugated coordination of organic linkers and the metal cations, over the formation of metal-oxo clusters^7^. The generation of controlled and coordinated reactive active sites, over the molecular scaffolds is highly essential. The main challenge associated with the MOF is to provide enhanced accessible active sites, low coordination number, modification in the local structure of the moieties, and low activity^8^. To overcome the above-mentioned limitations, the decoration of the inner pores of MOFs using the metal centers enhances the photocatalytic activity and stability^9^.

Ti-MOF is considered to be a complex containing the transition metals leading to the formation of active sites and metal-redox centers^10–12^. The charge transfer among the linkers to titanium-oxo-clusters, where the holes react with the sacrificial reagents^13^. Metal complexes, which possess metal atoms bridged with the ligands, are majorly employed to facilitate charge transfer and delocalization^14^. The development of various Ti-MOF-based materials synthesized through the 2-amino-terephthalic acid, with either Ti loading or Ru, Pt loading confers the linkers with the Ru centers or the Ti ions acting as either semiconducting materials, photosensitizers, or the cocatalysts^15–17^. Modification and variation of temperature through the annealing process concerning the reaction kinetics highly determine the stability, decomposition kinetics and higher electron transfer among the moieties and pendant groups at certain temperatures determining the deviation in the structural confirmations and the π–π stacking among the electro-active pendant groups^18^. The loading of Cu metal over the NH_2_-MIL-125(Ti) leads to a photocatalytic hydrogen generation of 490 µmol g^−1^ h^−1^^18^, whereas the loading of Ru over the NH_2_-MIL-125(Ti) leads to 426 µmol g^−1^ h^−1^^19^, ruthenium complex over Ti-MOF resulted in an activity of ~11.5 µmol. The development of the various NH_2_-MIL-125(Ti) with a variety of facets reveals that the (111) facet resulted in the superior activity of 60.8 µmol g^−1^ h^−1^^20^.

The initial report of the conjugated polymer-based photocatalyst was reported in the late 1980's, using poly(*p*-phenylene), which generates hydrogen through water splitting by using triethylamine^1^. Various attempts to rationalize the activity trends of the modified molecular entities over the hydrogen generation activity have been thoroughly determined using characteristics of hydrogen generation^21^, and spectral absorption^22^. The polymer system immersed over the aqueous media majorly includes the usage of the sacrificial electron donors. The structural tunability and electron in the usage of polypyridyl-based ruthenium complexes with modification over the pendant moieties to construct polymers that are photophysical and photoelectrochemically stable^21^. Several strategies have been deployed for the synthesis such as ATRP, RAFT, NMP, etc.^21–23^, which can be highly helpful as precursors or cocatalysts, sensitizers for the construction of nanomaterials that enhance light absorption and modify the photophysical properties. The composites are highly cost-effective and the sensitization of polymeric sensitizers over the interactive sites of Ru and Ti-MOF leads to intermolecular hydrogen bonding over the moieties leading to enhanced hydrogen generation activity.

Here we report a series of structurally modified conjugated polymers of Ru-phenanthroline doped Ti-MOF-based photocatalytic system for visible-light-induced hydrogen generation. The natural affinity of transition metal-based MOF and their doping using the polymer-based sensitizer is highly subjected to enhance the photocatalytic hydrogen generation activity. The Ru-polymer acts as a sensitizer on controlled loading over the Ti-MOF, as visualized P4T yielded superior hydrogen generation activity of 2438 µmol g^−1^ h^−1^, TON (9750), and AQY (5.33%) compared to the three armed and monomer determining that the increased chain, steric hindrance and helps in enhancing the activity and the effect of the defect and annealing over the composites have been carried out in the present charge-dynamic studies.

## Results

### Structure analysis and characterization

**Fig. 1:**
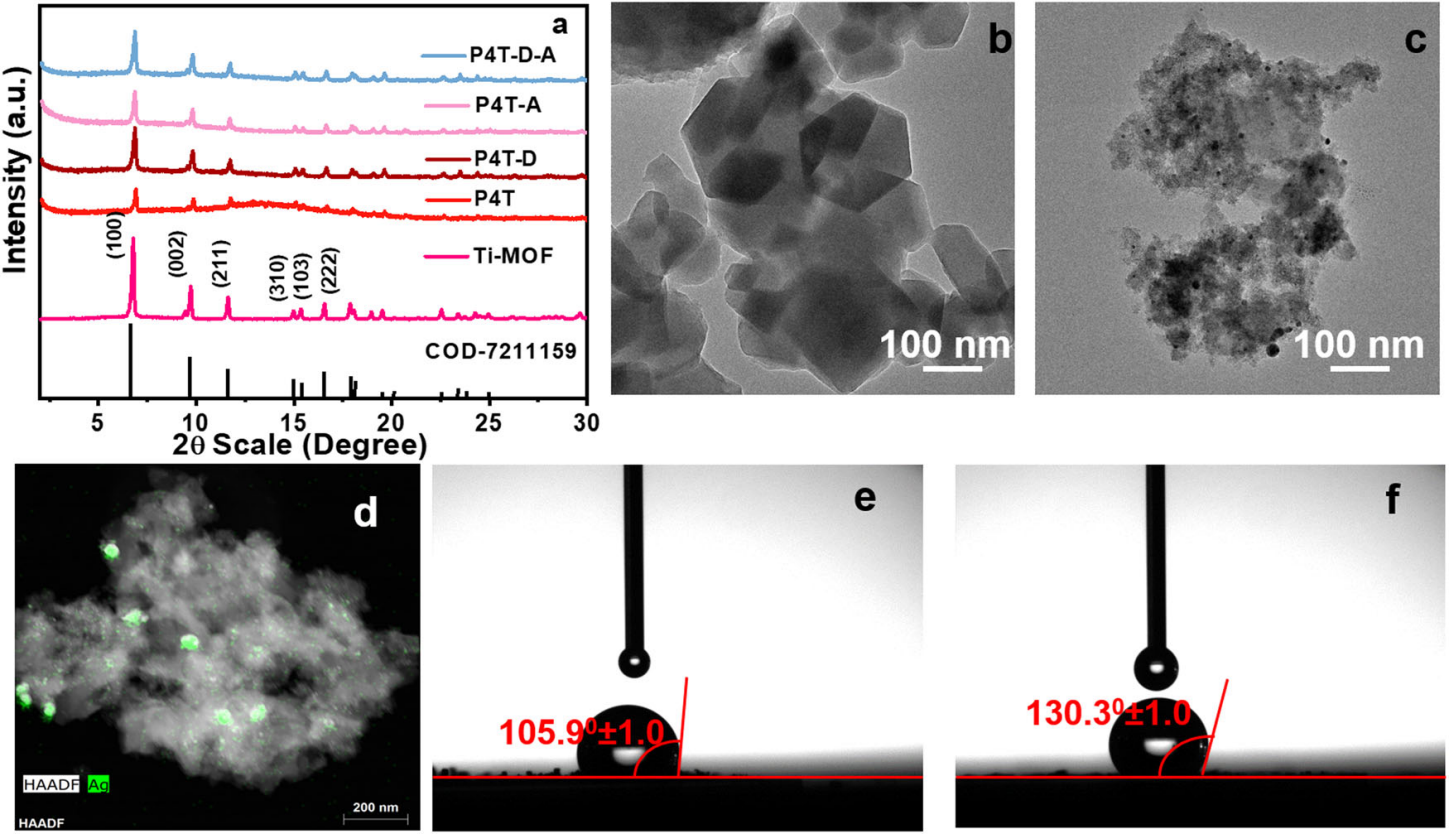
Structural studies determining photocatalyst elemental compositions. **a** XRD analysis of Ti-MOF, P4T, P4T-D, P4T-A, P4T-D-A, TEM analysis of **b** P4T, **c** P4T-D, **d** HAADF of Ag in P4T-D, and contact angle studies of **e** P4T, and **f** P4T-D.

The composites such as NH_2_-MIL-125 (Ti), PPDAR-4@Ti-MOF, PPDAR-4@Ti-MOF-D, PPDAR-4@Ti-MOF-A, PPDAR-4@Ti-MOF-DA are represented as Ti-MOF, P4T, P4T-D, P4T-A, P4T-D-A, respectively. The P4T subjected to annelation is termed as P4T-A, and that of the defect-rich P4T-D on annealing is termed P4T-D-A. A series of highly pure polymer-loaded MOFs were synthesized using a simple solvation method^22^. X-ray diffraction (Fig. 1a, Supplementary Fig. 1a, b) showed evidence of the polymer loading and annealing effect over the crystallinity and modifications in the π–π stacking over the compounds which creates a shred of evidence over the 15–20° which prioritizes the diffusion in the nano-segregation and the steric hindrance over the ruthenium center decreasing the isosteric heat retention leading to increase in the photocatalytic hydrogen generation efficiency over the annealing conditions^23–25^. The XRD peaks of the Ti-MOF show the planes of (101), (002), (211), (310), (103), and (222), which are in accordance with the reported NH_2_-MIL-125(Ti) structure which replicates the COD-7211159 file^24^, and the morphological variation studies carried out result in the octahedron morphology which are completely modified after exposure to the annealing and defect conditions (Supplementary Fig. 1b). The SEM and TEM analysis suggest a uniform crystallinity and pore structure depicting that the structure has been retained over the P4T and P4T-D composites (Fig. 1b, c, Supplementary Fig. 2c, i) and their respective annealing and defect creations shown in Supplementary Fig. 2d–i. The cubic morphology of the composites (Fig. 1b, c) highly determines the structural features of the moieties, which have been visualized and are in co-ordinance with the SEM and AFM studies.

The HAADF images determine the presence of the Ru, Ti, and O elements over the composites both in P4T and P4T-D, respectively (Supplementary Fig. 3a–k). The *d* values of 1.1 and 1.6 nm include the presence of (110) and (101) phases of Ti-MOF and Ru-centers, respectively. The dark particles are majorly due to the leftover silver particles which remained during the synthesis of defect-rich MOF (Fig. 1c, d). The defect engineering of the Ti-MOF has been visualized (Fig. 1c) where the porosity and the presence of the individual metal centers are determined and formulated to depict the efficiency and efficacy of the hydrogen generation activity with the primary effect on the light absorption capacity. The energy-dispersive X-ray spectroscopy (EDX) mapping shows the presence of Ru, Ti, and O (Supplementary Fig. 3e, k). The contact angles obtained were 105.9° ± 1.00° and 130.3° ± 1.00° for P4T and P4T-D, indicating the hydrophobic nature of the composites and the surface interfacial charge separation and surface interactions between the water molecules and the catalysts as shown in Fig. 1e and f, the increase in the hydrophilicity is observed over the P4T than over the defect incorporated composite. The creation of defects has increased the hydrophobicity of the compound as shown in Fig. 1e and f.

**Fig. 2:**
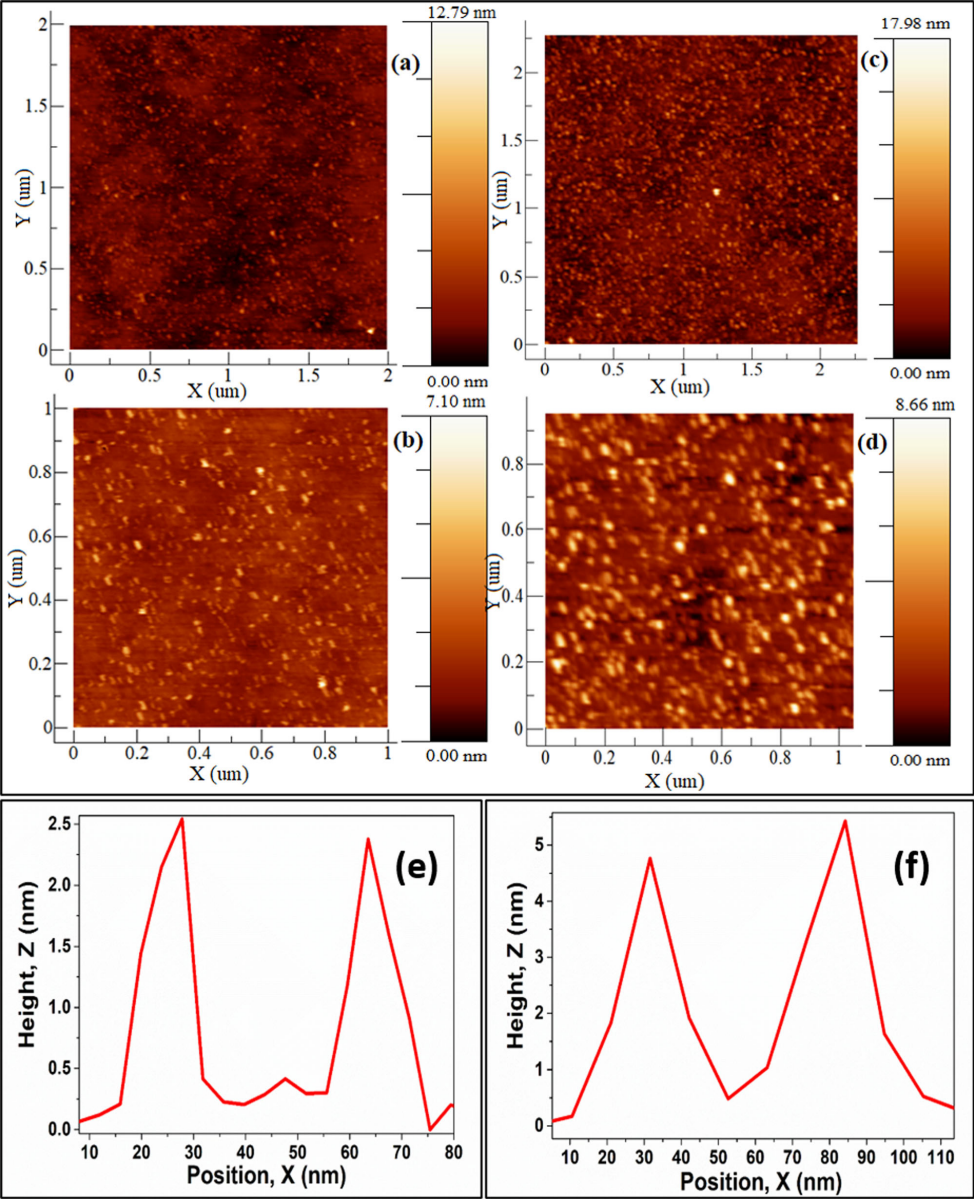
AFM analysis determining the structural roughness. Tapping mode two-dimensional AFM images of **a**, **b** P4T, **c**, **d** P4T-D, the apparent height and diameter of isolated nanoparticles of **e** P4T and **f** P4T-D.

The changes in the surface morphology and the root mean square (RMS) roughness are examined by atomic force microscopy (AFM). Tapping mode AFM has been carried out for the samples of P4T and P4T-D. Figure 2a-d shows the 2D AFM images of P4T and P4T-D [Fig. 2e-f and Supplementary Fig. 5]. Morphology showed tiny nanostructured particles covering throughout surface area, however, the apparent length and height of this nanoparticle are 15 & 2.4 nm for P4T (Fig. 2e) and 40 & 5 nm for P4T-D (Fig. 2f). Comparatively, P4T has a small nanoparticle size to that of P4T-D, which leads to reduced RMS roughness. The lower RMS roughness for the P4T (0.4743) compared to P4T-D (1.157) reveals the enhanced short circuit current density (*J*_sc_), as shown in Supplementary Fig. 5^26^. It affects the optical properties and significantly rises the photocatalytic performance of solar hydrogen. AFM studies reveal that the presence of the different metals doesn’t alter the general nature of the film morphology but the creation of the defect over the existing anchoring groups has led to the agglomeration of the composite and the deviation in the morphology has been visualized in Fig. 2^26^.

The Fourier transformation infrared spectroscopy (FTIR) of P4T and P4T-D with different PPDAR and their annealing effects at 180 °C resulted in the modification and extended elongation over annealing and degradation or decarboxylation over the defect-rich moieties as visualized over the Ti-MOF-D which has been shown in Supplementary Fig. 4d, e. The peaks at 770, 1529, and 3100 cm^−1^ of the composites reveal the interaction between the Ti-MOF and PPPDAR moieties corresponding to Ru–N, C–O, and C=O, respectively^16–18^. The peak at 1650 cm^−1^ is due to the presence of amine group in the Ti-MOF, which has been shown in Supplementary Fig. 4d, e, and the defect effect over the region of 900–1750 and 2500–3700 cm^−1^ in comparison with that of the Ti-MOF(D) and PPDAR@Ti-MOF composites have been visualized over the defect induced and the annealed moieties depicting the interplanar π–π stacking over the moieties (Supplementary Fig. 4d, e).

### X-ray absorption spectroscopy (XAS) measurement

**Fig. 3:**
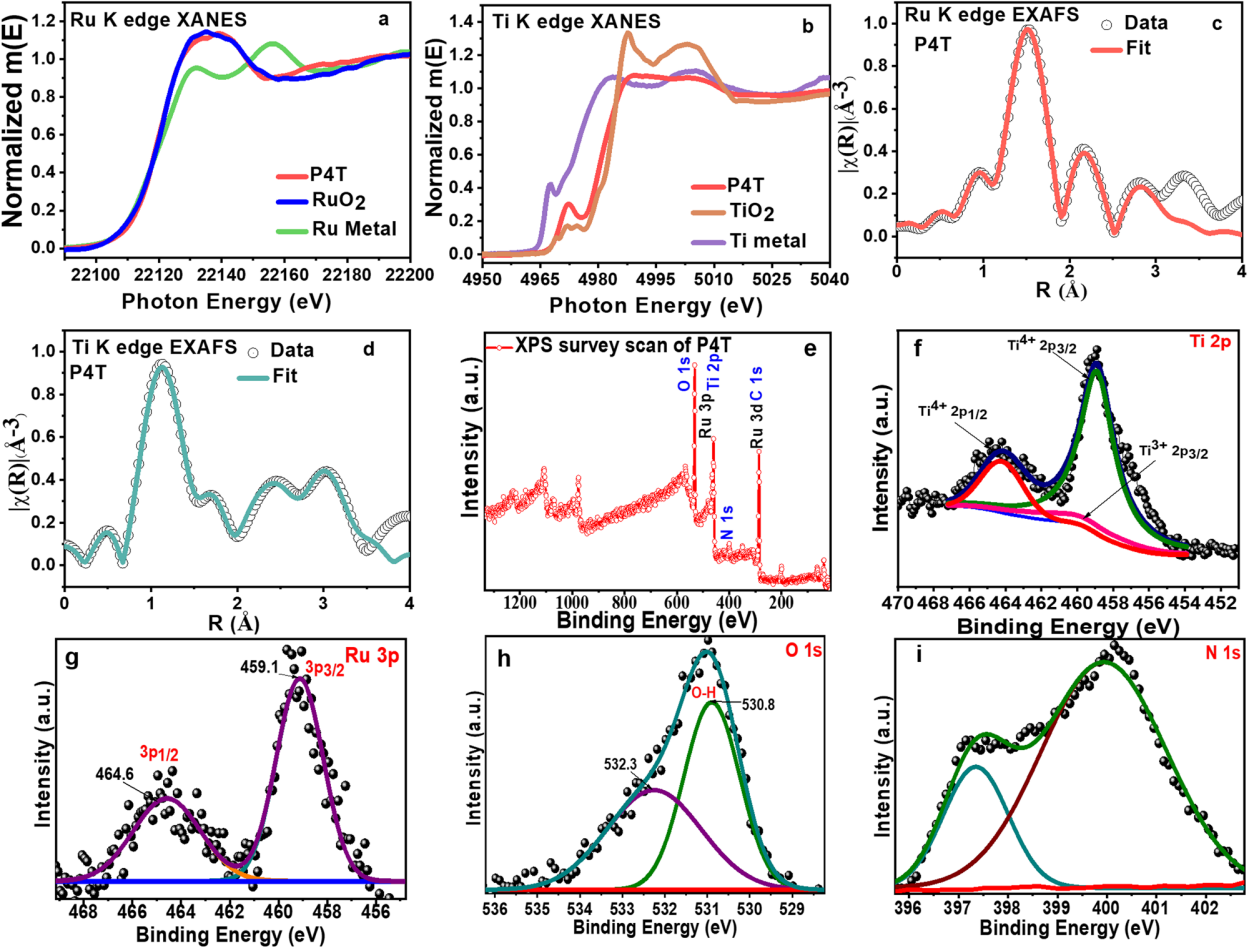
EXAFS and XPS analysis of the synthesized photocatalysts. EXAFS spectra **a** Ru K edge, **b** Ti K edge, fitting edges of **c**, **d** P4T cluster, XPS spectra of P4T, **e** Survey, **f** Ti 2*p*, **g** Ru 3*d*, **h** O 1*s*, and **i** N 1*s*.

Figure 3a, b shows the XANES spectra of the P4T sample at Ru K edge, and Ti K edge respectively in comparison with the reference sample spectra. The analysis of the EXAFS spectra is carried out using the standard reported procedure^27–30^. Figure 3c shows the $\chi (r)$ vs. *r* plots of the P4T sample at the Ru K edge. The experimental $\chi (r)$ vs. *r* data of the P4T sample is fitted over 1–3.0 Å assuming a Ru–O shell, a Ru–Ru shell, and a Ru–Ti shell. The coordination numbers, distances, and disorder factors of all shells have been varied on fitting with the obtained experimental data. Ru K edge EXAFS fitting results have been tabulated in Table 1. The versus plot of the P4T sample at the Ti K edge has been shown in Fig. 3d and the data has been fitted from 1 to 3.5 Å assuming two Ti–O shells and two Ti–Ti shells. The Ti K edge EXAFS fitting is shown in Table 2. The Ru–O/N coordination (2.07 Å) and the Ru–Ti (3.11 Å) indicate that extended bond length over the Ru–Ti in the composite results in the incorporation of the Ru metal over the MOF structure and does not replace any Ti centers.

**Table 1:** Ru K edge EXAFS Fitting results.

	P4T		
	*R* factor = 0.0066		
	*R* (Å)	*N*	*σ*^2^ (Å^2^)
Ru–O/N	2.07 ± 0.01	2.4 ± 0.2	0.0022 ± 0.0011
Ru–Ru	2.77 ± 0.02	2.8 ± 0.5	0.0098 ± 0.0030
Ru–Ti	3.11 ± 0.02	2.4 ± 0.8	0.0101 ± 0.0023

**Table 2:** Ti K edge EXAFS Fitting results.

	P4T		
	*R* factor = 0.0122		
	*R* (Å)	*N*	*σ*^2^ (Å^2^)
Ti–O	1.72 ± 0.01	3.3 ± 0.2	0.0138 ± 0.0011
Ti–O	1.92 ± 0.01	1.3 ± 0.1	0.0045 ± 0.0017
Ti–Ti	2.99 ± 0.01	1.3 ± 0.1	0.0011 ± 0.0009
Ti–Ti	3.77 ± 0.01	2.1 ± 0.3	0.0045 ± 0.0018

The XPS spectra in Fig. 3e-i, and Supplementary Fig. 3l, show the positive binding energy of N 1*s* over the 402.3 eV^31^, depicting the formation of the positively charged nitrogen species, the peak shifting over the 399.6 eV depicts the local electron distribution over the NH_2_ and the Ru-centers^19,32^. The peak at 529.6 eV corresponds to the Ti–O species, and the high-resolution Ru 3*d* and 3*p* centers lead to new bands at 283.4 and 458.7 eV corresponding to the Ru–N and Ru–O bond respectively (Fig. 3e–i)^23,33^. Figure 3f visualizes the presence of consistent Ti^4+^ states over the Ti-MOF lattice. The shoulder peaks were obtained over the 464.7 eV corresponding to the Ti2*p*1/2 and the peak over 458.9 eV to that of the Ti2*p*3/2. The slight shift in the values majorly depicts the influence of the Ru ions over the Ti lattice. The presence of the Ti^3+^ ions is also observed in the Fig. 3f, g but their intensity is low and the charge recombination with the Ru^3+^ ions is more favorable with the Ti^4+^ ions^34^.

### Positron annihilation lifetime spectra

The fitting of the positron annihilation lifetime spectra is majorly carried out to be fitted up to two components, Ti-MOF and Ti-MOF-D. As per the conventional procedures, the lifetime measurements are named *τ*_1_ and *τ*_2_ in the increasing order of magnitude and the corresponding intensities are called *I*_1_ and *I*_2_^35^. The lifetime and their consecutive intensities are tabulated in Table S1, the table reveals the presence of two positron lifetime components over the Ti-MOF indicates the presence of a partial defect, where the first component majorly corresponds to the bulk matrix whereas the second is due to the positron annihilation over the defects. Over etching, the lifetime has been increased marginally with intensity over an expense to that of the second component, this increase over the lifetime majorly constitutes the increase in defect size as shown in Supplementary Fig. 6. The results show that the larger defects have further grown in size with their number reducing besides the creation of smaller defects, indicating that some of the pre-existing defects have grown and coalesced and further some smaller defects have been created.

### Photophysical properties

**Fig. 4:**
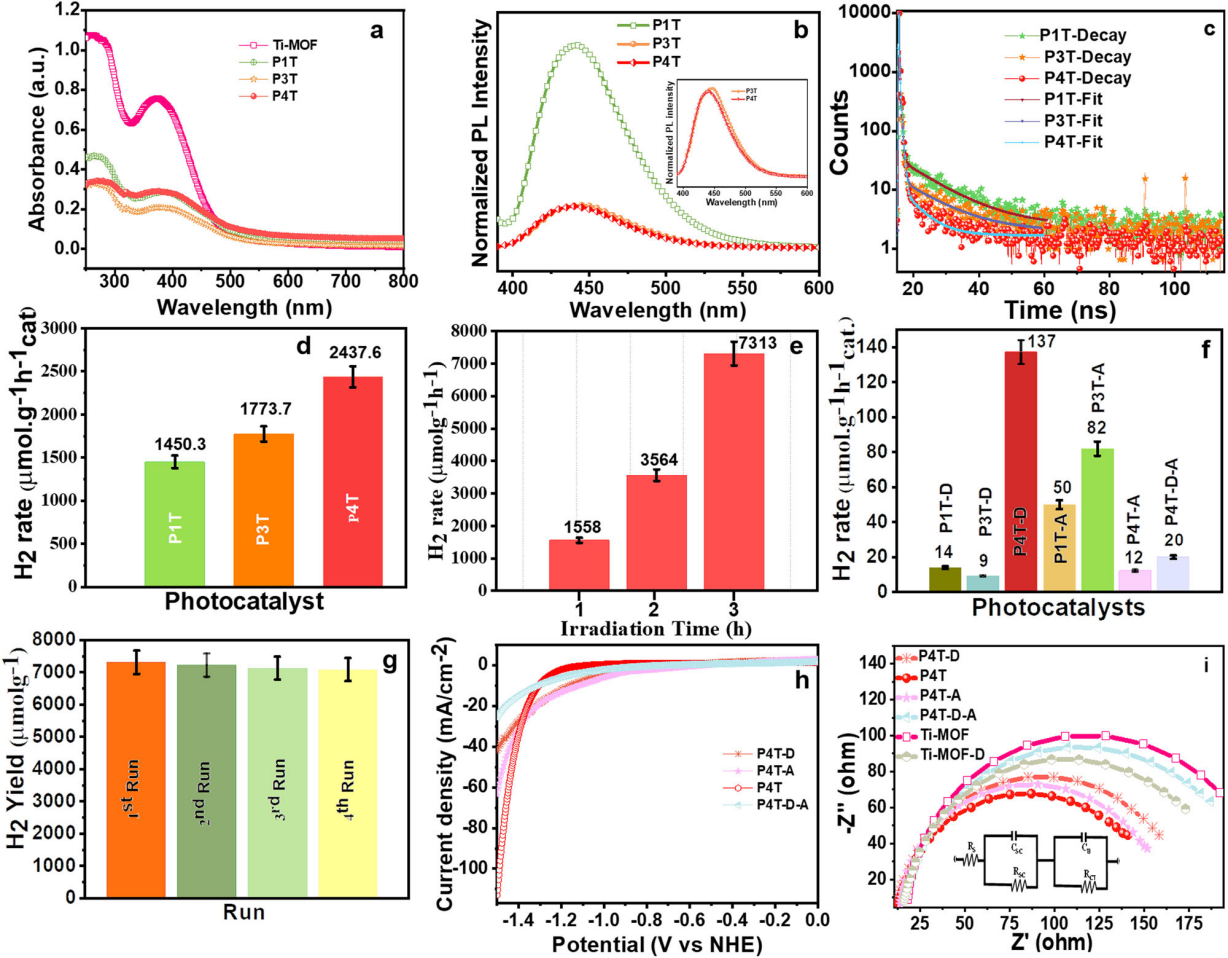
Photocatalytic and photoelectrochemical studies of the composites. UV–vis absorbance spectra of **a** Ti-MOF, P1T, P3T, P4T, **b** photoluminescence studies of the composites, **c** time-correlated single-photon count measurements, error bar diagram of **d** P1T, P3T, P4T, **e** time profile study of hydrogen production for P4T, **f** P4T-D, P1T-D, P3T-D, P1T-A, P3T-A, P4T-A, P4T-D-A, **g** error bar graph of recyclability studies, **h** linear sweep voltammetry studies, and **i** Nyquist plot of the PPDAR@Ti-MOF composites. (error bars: 5% of error has been considered so far).

The UV–vis diffuse reflectance spectra (DRS) of the synthesized photocatalysts have been visualized in Fig. 4a and Supplementary Fig. 4a. The absorbance spectra show that the pristine Ti-MOF exhibits a broad optical absorbance with the absorbance edge at 400 nm whereas the absorbance of the Ru-based polymer loaded Ti-MOF has resulted in a decline in the absorbance capacity which significantly determines that the porosity of the MOF has been occupied by the PPDAR moiety in comparison of P1, PPDAR-3 and PPDAR-4 shown in Supplementary Fig. 4b, c. This is in inter-relation with the BET analysis as shown in Table S2.

In addition to the bandgap of the titania moiety, a slight enhancement in the visible light region has been observed with the doping of the P1, PPDAR-3, and PPDAR-4. The results depict that the increase in the steric hindrance and the bulky moieties attachments in the polymer moieties have resulted in the higher tendency of light absorbance as visualized in the plot and their assigned band gaps are in the order P4T > P3T > P1T > Ti-MOF i.e., 2.36 > 2.4 > 2.45 > 2.57 eV, respectively, as shown in Supplementary Fig. 4b, c. The photoluminescence studies of the photocatalysts synthesized have been investigated as shown in Fig. 4b. The P1T photocatalyst results in higher emission of light indicating lower light absorption whereas the P3T and P4T possess a better absorption so the emission is quite near but the quenching effect of P4T is comparatively high based on the values obtained which highly favors the photocatalytic water splitting efficiency. The life measurements have been in high correlation with that of hydrogen production confirming that the P4T should possess increased charge-carrier and generation capacity as shown in Fig. 4c. The lifetime measurements displayed as 0.16, 0.061, and 0.31 ns of P1T, P3T, and P4T, respectively, and their photocatalytic activity has been correlated with the results as shown in Table S3. The electron quenching leads to increased electron–hole recombination dynamics which favors the hydrogen generation efficiency due to its increased electron-transfer properties. The extended lifetime enhances the electron stability over the excited state, leading to higher light absorption and enhanced water-splitting ability. The polymeric groups act as the fluorophores, where the dynamic quenching happens majorly during the decay process, the quenchers (enolate groups) deactivate the excited states of the fluorophores. So, the deactivated fluorophores follow the nonradiative decay processes, leading to faster decay and low lifetime measurements, herein P4T possesses a higher lifetime over the three-decay system of the non-radiative process indicating low-decay^36^. The three-exponential decay mechanism involves the generation of fluorescence, which is considered to be through the semiconductor exciton transition from the conduction band to the valence band.

Thermogravimetric analysis (TGA) of the composites has been shown in Supplementary Fig. 7a–c revealing the gradual weight loss for all the samples up to 150 °C, as shown in Supplementary Fig. 7a–c, P4T possesses higher thermal stability between 185 and 400 °C indicating higher stability which increases the photocatalytic activity. The N_2_ adsorption–desorption isotherm exhibit a Type-II pattern as shown in Supplementary Fig. 7d–i, which is quietly related to the microporous and mesoporous kinetic materials. The Brunauer–Emmett–Teller (BET) surface area and pore volume of Ti-MOF and Ti-MOF-D are 756.04 m^2^ g^−1^, 0.6692 cm^3^ g^−1,^ and 713.88 m^2^ g^−1^, 0.6274 cm^3^ g^−1^, respectively (Table S2). The superior pore volume, and mean pore diameter of the Ti-MOF concerning the Ti-MOF-D are consistent with the hollowness and interpretation of the pores as shown in Supplementary Fig. 7d, e, the narrow pore size of the composites has been visualized as shown in Supplementary Fig. 7f, g^37–39^. The surface area of the PPDAR-loaded MOF defect and non-defect resulted in a decline in the surface area with the PPDAR loading as shown in Supplementary Fig. 7f, g. The surface area of P4T-D is 133.454 m^2^ g^−1^, pore volume of 0.402 cm^3^ g^−1^, and diameter of 3.829 nm, whereas that of the P4T are 199.059 m^2^ g^−1^, 0.454 cm^3^ g^−1^, and diameter of 1.422 nm, respectively. The results envisage that the decrease in the surface area of the composites is due to the deposition of the PPDAR materials over the Ti-MOF and Ti-MOF-D which is majorly due to higher molecular weight occupying the total surface of the pores thereby decreasing the surface area and enhancing the light absorption capacity of the materials. The adsorption isotherms of the other samples have also been included, which confirm the low porosity, and activity of the composites as shown in Supplementary Fig. 7h, i. The electrostatic hindrance and the electron–hole recombination kinetics of the composites support hydrogen production and BET data is in synergy with the results.

### Photocatalytic hydrogen generation activity

As shown in Fig. 4d–g, Supplementary Fig. 4f–h the superior photocatalytic hydrogen generation efficiency has been observed from the P4T composite containing more number of ruthenium centers and electron donor groups to enhance the activity^25^, whereas P4T-D and P4T-A, P4T-D-A exhibited lower hydrogen generation efficiency along with P1T and P3T. P4T series generate 17 folds (2348 µmol g^−1^ h^−1^) increasing hydrogen generation over the other composites. In addition to the hydrogen generation rate, the apparent quantum yield (AQY) is 5.33% and 0.30% for P4T and P4T-D, respectively. The MOF without the polymeric PPDAR-loading results in lower efficiency. The effect of annelation is thoroughly investigated and the results reveal that the effect of temperature over the steric hindrance is highly affected when loaded over the surface of the Ti-MOF. The higher steric hindrance over the P4T-A resulted in low hydrogen generation efficiency due to their structural deviations and bond cleavages in comparison to the other P1T-A and P3T-A. The results are shown in Fig. 4f, Supplementary Fig. 4f determines the effect of the defect creation and annealing drastically decreases the hydrogen generation activity and sterically hinders the electron transfer over the moieties. The stability and recyclability studies determine the sustained hydrogen generation activity of the P4T after 4 runs (see Fig. 4g and Table S4), indicating superior and excellent stability, which was further confirmed through the TGA, photoelectrochemical, and XPS analysis. The obtained report is 5 folds higher than the benchmark report which is tabulated in Table S4. The graph of TON vs. photocatalysts showed in Supplementary Fig. 4g, h, confirms the higher hydrogen efficiency of P4T in comparison to all the other synthesized materials.

### Photoelectrochemical studies

To probe the redox kinetic processes of the P4T composite and the initial PPDAR moieties along with Ti-MOF^40,41^, independent cyclic voltammetry (CV) studies have been carried out over the 0.1 M Bu_4_NClO_4_ aqueous solution. The CV scans tend to possess two reversible redox peaks at −1 and +1.2 V corresponding to the reversible Ti^3+^/Ti^4+^ redox process (see Supplementary Fig. 8a–i). In comparison, the P4T and P4T-A possess three peaks at −0.92, +0.54, and +1.02 V, and that of the P4T-D-A a single reduction peak at +1.05 V (vs. SCE). The modification of the voltage values over the loading of different polymers has resulted in various new voltage values. The results envisage that the PPDAR molecules possessing the Ru metal tend to the generation of multiple anode–cathode peaks at 0.8–0.9 mV due to the redox coupling between the Ru and the material. For a better understanding of the linear sweep voltammetry (LSV) of the composites carried at a scan rate of 2 mV s^−1^ from −1 to 1 V vs. SCE and the Ti-MOF (see Fig. 4h, Supplementary Fig. 9) and Nyquist plots have also been performed (see Fig. 4i). The much smaller arc of the P4T than that of the other composites suggests the P4T possesses improved charge-transfer kinetics (see Fig. 4i), and consistent electron–hole recombination kinetics which has been verified by the photoluminescence and lifetime measurement studies. The photocurrent studies of the Ti-MOF and Ti-MOF-D tend to possess superior stability and enhanced charge-separation efficiency as shown in Supplementary Fig. 4i.

### Density functional theory studies

**Fig. 5:**
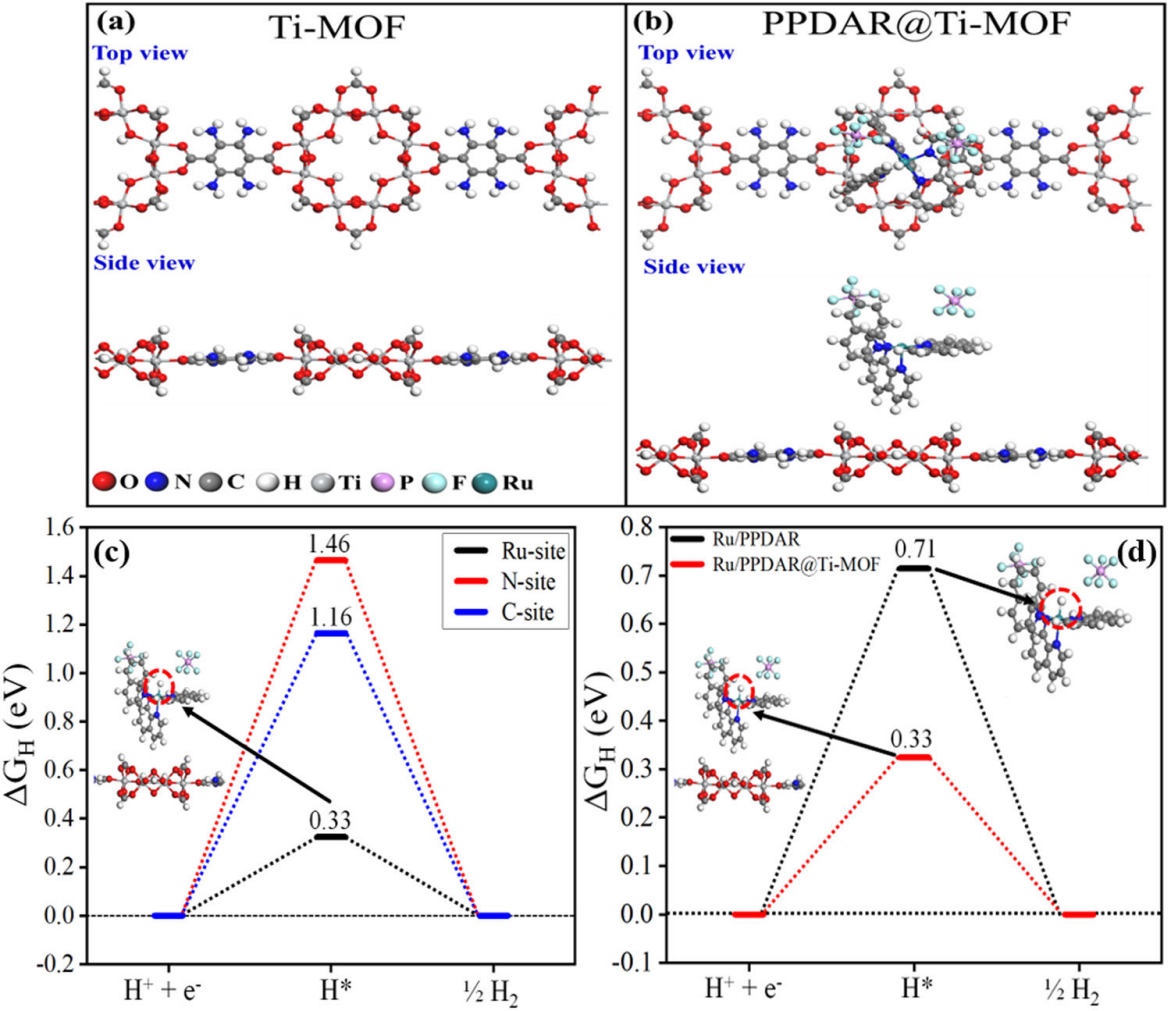
DFT analysis of the ruthenium polymer grafted Ti-MOF photocatalysts. DFT-optimized ball and stick structures of **a** Ti-MOF, **b** PPDAR@Ti-MOF with their top and side view, **c** hydrogen binding free energy (ΔG_H_) at ruthenium, carbon, and, nitrogen sites. Adsorbed site is given in the inset marked with a red dotted circle. We have only shown the structure around the polymer, and **d** hydrogen binding free energy (ΔG_H_) at the ruthenium site of polymer (PPDAR) with and without Ti-MOF. The inset indicates structure with and without MOF. Ru-sites are marked with a red dotted circle. We have only shown the structure around the polymer.

We have also employed density functional theory-based calculations to evaluate the hydrogen binding free energy (ΔG_H_) for investigating the role of PPDAR polymer at Ti-MOF in the catalytic activity of the HER. ΔG_H_ of hydrogen adsorption is an excellent descriptor to define the catalytic activity of a reaction on the surface/sites and it should be neither too high nor too low^34^. Optimized structures of Ti-MOF and PPDAR@Ti-MOF which were used to identify the intrinsic properties and reactivity of the surface are given in Fig. 5a (see computational details to check the method). Firstly, we calculated ΔG_H_ on different sites of the PPDAR unit after hydrogenation. There is a difference in ΔG_H_ when different sites are hydrogenated. Interestingly, after removing a benzene ring from the PPDAR unit, the ΔG_H_ value at the nitrogen and carbon site (Fig. 5b) is about 1.46 and 1.16 eV, respectively, which indicates a higher overpotential. Whereas, the ΔG_H_ value at the Ru site decreases up to 0.33 eV (Fig. 5c), showing that the removal of atoms from Ru polymer provides an additional charge at the Ru site which leads to stronger hydrogen adsorption and favors the energetics towards H_2_ formation. These changes also indicate the presence of *d*-orbitals of Ru, favorable for improving the catalytic activity of the polymers. Further, to check the role of Ti-MOF incorporated with Ru polymer (PPDAR), we have also calculated the ΔGH on the Ru site of polymer with Ti-MOF and without Ti-MOF (see Fig. 5c). Results indicate that when the hydrogenation takes place at the Ru site of polymer without Ti-MOF, ΔG_H_ is found to be 0.71 eV, whereas, with Ti-MOF, it is significantly reduced to 0.33 eV, which suggests that the Ru polymer incorporated Ti-MOF is more stable and further improves the efficiency of the systems for photocatalysis (see Fig. 5d). The NMR data envisages the formation of the PPDAR moieties (see Supplementary Figs. 10 and 11).

**Fig. 6:**
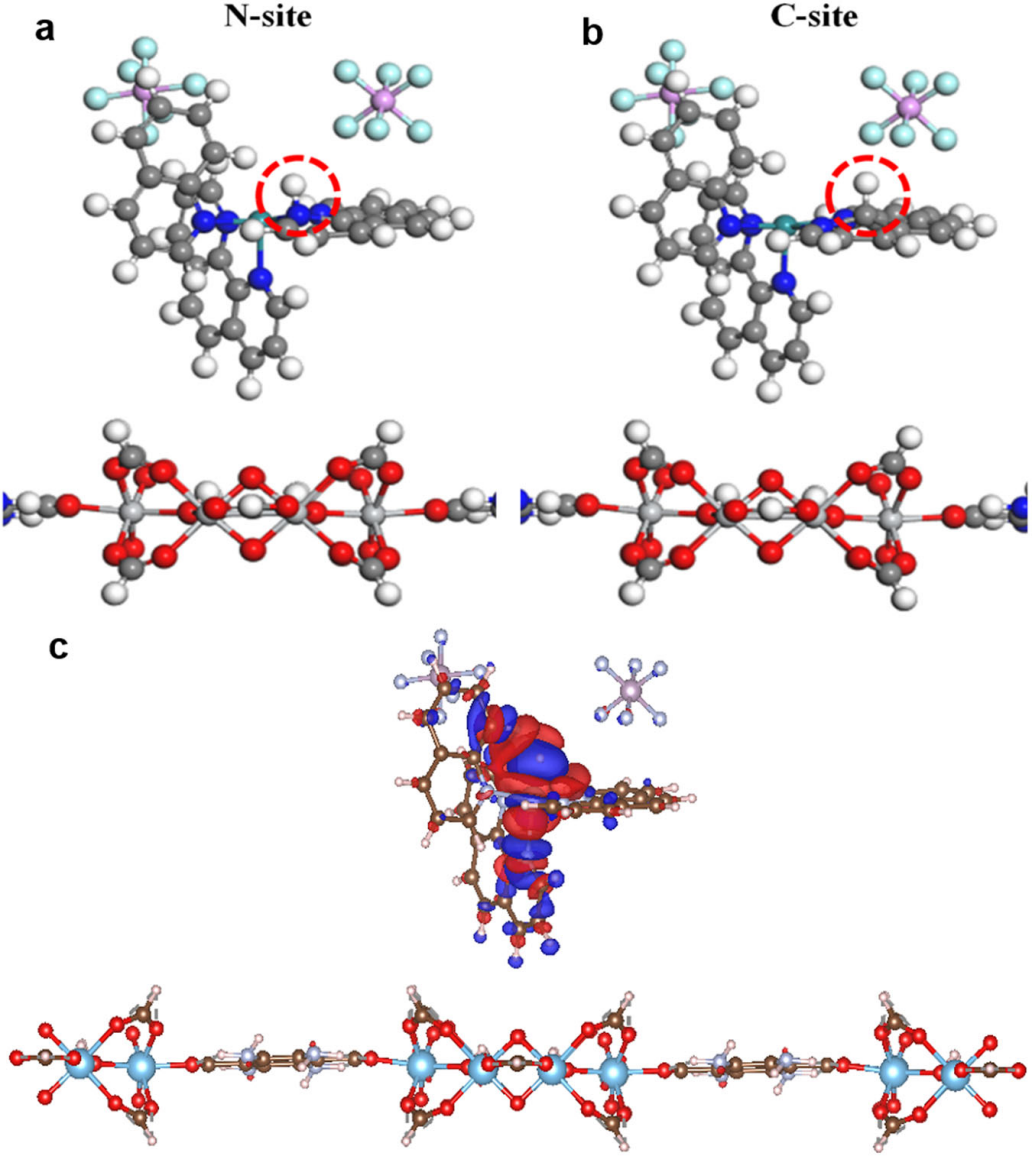
DFT studies and their consecutive models. DFT-Optimized ball and stick structures of hydrogen adsorption on **a** N-site, **b** C-site of polymer. We have only shown the structure around the polymer, **c** difference charge density of PPDAR@Ti-MOF with the adsorption of hydrogen, where the iso-surface value is set to be 0.0006 e Å^−3^, and the positive and negative charges are shown in red and blue.

The HOMO–LUMO levels of the polymers enhance the electron transfer capacity and higher light absorption for the generation of electrons and the transfer to the semiconducting material for water splitting. The triethanolamine (TEOA) being a sacrificial electron donor (SED) enhances the charge separation and electron–hole recombination criteria to enhance efficiency. The HOMO–LUMO values and diffusion coefficient (*D*0) values are in an order uniform to all the studies confirming the fast electron transfer through the polymer surface to the semiconductor surface where the polymer on the absorption of light the electron gets excited from HOMO to LUMO thereby transferring an electron to the surface of the Ti-MOF for water reduction see Fig. 6a–c. The electron–hole recombination kinetics has been further reduced through the usage of the TEOA as a sacrificial electron donor.

**Fig. 7:**
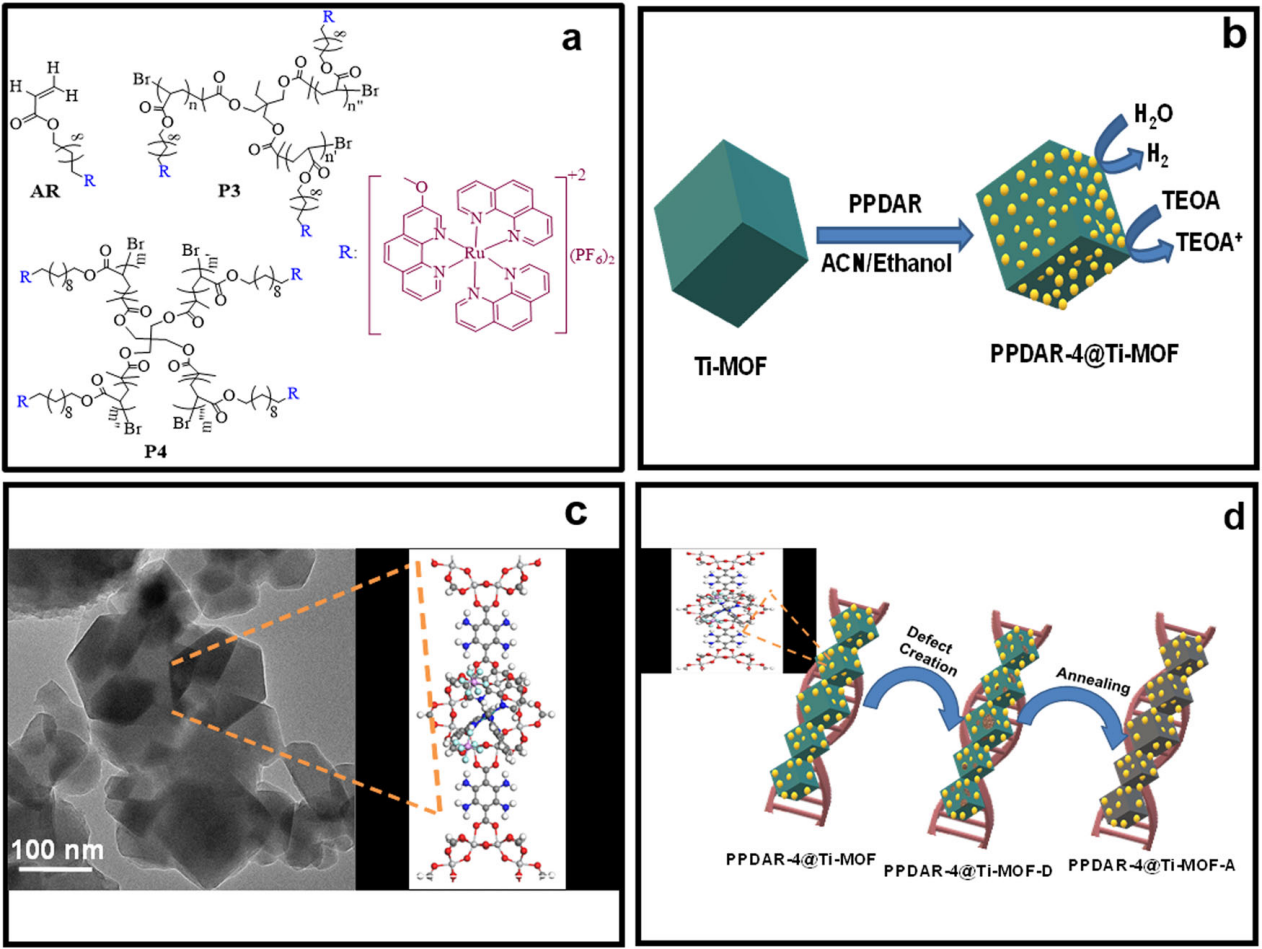
Chemical structures and mechanistic pathway. **a** Chemical structures of the acrylate ruthenium (AR or P1) complex, 3-armed AR polymer (PPDAR-3) and 4-armed AR polymer (PPDAR-4), **b** mechanistic pathway for hydrogen production, **c** TEM with the structure image of Ti-MOF, and **d** Schematic view of the modification conditions.

The structural representation of the PPDAR moieties has been shown in Fig. 7a. The photocatalytic hydrogen generation rate of the PPDAR moieties follows a passion of P4T > P3T > P1T. The superior activity of P4T is found to be due to the enhanced interactions between the electro-active pendant groups and Ti-MOF and improved π–π stacking of the pendants, which is envisaged from the hydrogen production analysis obtained so far^40^. During a photocatalytic cycle, the photogenerated electrons were transferred from the linker moieties towards the ruthenium centers forming highly active Ru^3+^/Ti^4+^ mixed metal centers. The increase in the number of pendant groups in the PPDAR resulted in an increasing trend of hydrogen generation due to the agglomeration over the surface, availability of the active sites, steric hindrance and the covalent interactions among the moieties following the trend P4T > P3T > P1T. This alternative modification in the structure through Ru metal centers-based photosensitization leads to highly enhanced charge dynamics and a prolonged lifetime which synergistically enhances the photocatalytic hydrogen generation, using ruthenium-based polymer, as photosensitizers (Fig. 7b).

The photocatalytic HER cycle follows a schematic electron transfer according to the order as below (Fig. 7b): The light irradiation over the photosensitized polymer, through the excitation donates electrons to the conduction band of the semiconducting material where the electron changes the state of the Ti (III) leading to the formation of the protons and Ti (IV) formation. The Ti (IV) accepts electrons from the reduced polymer Ru (III) center forming a Ti^4+^/Ru^3+^ bond and the usage of sacrificial electrons which donates electrons to improve the charge-recombination dynamics and the stability of the material with a continuous flow of electrons from SED for hydrogen generation.

The effective shielding of the Ru (III) centers results in charge transfer quenching, increased diffusion coefficient value of PPDAR-4 confirms the fast electron transfer in the semiconductor electrode surface and thereby enhances the water reduction process. The π–π* electrostatic interactions^17^, between the pendant groups and the Ti-MOF led to the donation of the electrons for the water splitting and increased green fuel generation. The schematic representation of the photocatalyst synthesis and the TEM representation in Fig. 7c and their modification over the different annealing and defect creation is shown in Fig. 7d.

## Discussion

In comparison to all the experimental as well as theoretical calculations, the P4T composite resulted in superior hydrogen generation activity which is proven so far. The increase in the H_2_ activity is majorly due to the steric hindrance as well as the hydrophilic property of the composite. The ΔGH values for HER are supported by the overpotential values in the *J*–*V* curve of the dye moieties. High photocurrent activities confirm the reversible reactions and electron transfer over the Ti^4+^ ions and Ru^3+^ centers. The role of the defect creation, and annealing conditions are further studied and the results reveal that the distortion in the morphology has resulted in low H_2_ activity after defect creation and annealing of the composites.

In summary, we report for the first time, the development of phenanthroline-based ruthenium polymer (PPDAR) incorporated Ti-MOF light-harvesting system. The regular arrangement over the Ti-MOF is modified after the loading of Ru-polymer, which was confirmed through various characteristic studies. High exposure of active sites caused majorly due to the loading of PPDAR polymer resulted in superior activity. The steric hindrance, nano-aggregation, the effect of conferring the organic linkers, and the replacement of the transition metal centers, and their activity over the defect-creation and annealing conditions have been demonstrated and believed to be a key factor in their relative photocatalytic hydrogen generation activity. The incorporation of Ru^3+^ over the Ti^4+^ ions in MOF structure, their increased interactions among the electro-active pendant groups, hydrophilic properties, enhanced charge-separation, and nano-segregation over the polymeric chains of the Ru-polymer resulted in 17 folds superior hydrogen generation efficiency of the P4T complex. The lower roughness, reduced ΔGH values, and high photocurrent activities confirm the reversible reactions and electron transfer over the Ti^4+^ ions and Ru^3+^ centers. This study provides a new strategy to enhance the performance of Ti-MOF and the grafting of the catalytic photosensitizer ligands in solar energy conversion.

## Methods

### Materials and reagents

High-purity materials of analytical grade were procured from Sigma Aldrich, the USA without any purification. 2-Aminoterephthalic Acid, Acetonitrile, Ethanol, Titanium (IV) isopropoxide (TTIP), Tetrabutylammonium perchlorate (Bu_4_NClO_4_), Dimethyl Formamide (DMF), AgNO_3_, Methanol, and K_2_S_2_O_8_.

#### Preparation of the photocatalysts

##### Synthesis of Ti-MOF

Ti-MOF has been synthesized using a simple solvothermal process^21^. The typical procedure includes the addition of 2.4 mL of TTIP and 2.2 g of 2-aminoterephthalic acid into a suspension of DMF:methanol mixture (36:4 mL). The obtained suspension was transferred into a 100 mL stainless steel Teflon-lined autoclave and subjected to hydrothermal treatment at a temperature of 150 °C for a time period of 48 h. The filtrate has been collected through centrifugation by washing it using methanol to remove the excess DMF present in it. Finally, the resultant product was dried at 70–80 °C.

##### Synthesis of Ti-MOF-D

The synthesis of Ti-MOF-D has been carried out through the silver-catalyzed decarboxylation approach^10,20^. Typically, equivalent weights of 25 mg have been taken of AgNO_3_, and K_2_S_2_O_8_ along with the 150 mg of Ti-MOF was evenly dispersed in 20 mL of Acetonitrile (ACN) which was subjected to sonication for a period of 10 min, and the resultant mixture has been preheated in a silicon oil bath at 150 °C for 60 min. After the completion of the reaction, the resulting product is transferred into ice water for quenching and to prevent further decarboxylation etching. The obtained product was washed with deionized water and collected through the centrifugation technique followed by drying at 70 °C overnight.

##### Synthesis of PPDAR/Ti-MOF

The synthesis of the PPDAR moieties has been carried out using the reported procedure^23^. Wherein, the synthesis of the PPDAR/Ti-MOF follows a typical dye sensitization procedure^42^. Initially, 0.5 µmol per 100 mg of the dye sample was dispersed in a solution of 1:1 Vol/Vol of Ethanol and acetonitrile, and to this 100 mg of the Ti-MOF is added and subjected to ultrasonication for 10 min followed by stirring under dark conditions for 24 h. The obtained solution was washed and centrifuged with water and ethanol and dried at 70 °C to separate the solvent and the product was obtained which is stored in dark conditions to avoid light sensitization and labeled as P1, PPDAR-3, PPDAR-4@Ti-MOF and P1, PPDAR-3, PPDAR-4@Ti-MOF(D), respectively.

##### Annealing of PPDAR@Ti-MOF composites

The annealing of the photocatalysts has been carried out using the vacuum overheating temperature of 180 °C for 2 h under the vacuum conditions^27^, and further subjected to nitrogen flow to maintain an inert atmosphere for a period of 1 h and is allowed to cool to room temperature the slight modification in the color has been observed which are labeled as P1T-A, P3T-A, P4T-A, P4T-D-A, respectively.

All the characterization details are provided in the Supplementary Methods in the Supplementary information. X-ray absorption spectroscopy measurements. X-ray absorption spectroscopy (XAS) measurement of the P4T sample has been carried out at Ru and Ti K edge in fluorescence mode at the Scanning EXAFS Beamline (BL-9) at the Indus-2 Synchrotron Source (2.5 GeV, 200 mA) at the Raja Ramanna Centre for Advanced Technology (RRCAT), Indore, India^28,29^. The beamline uses a double crystal monochromator (DCM) which works in the photon energy range of 4–25 keV with a resolution of 104 at 10 keV. A 1.5 m horizontal pre-mirror with meridional cylindrical curvature is used before the DCM for collimation of the beam and higher harmonic rejection. The second crystal of the DCM is a sagittal cylinder with a radius of curvature in the range of 1.28–12.91 m which provides horizontal focusing to the beam while another Rh/Pt-coated bendable post mirror facing down is used for vertical focusing of the beam at the sample position. For measurements in the fluorescence mode, the sample is placed at 45° to the incident X-ray beam and the fluorescence signal (${I}_{{\rm {f}}}$) is detected using a Si drift detector placed at 90° to the incident X-ray beam. An ionization chamber detector is used before the sample to measure the incident X-ray flux (${I}_{0}$)) and the absorbance of the sample $\left(\mu =\frac{{I}_{{{{{{\mathrm{f}}}}}}}}{{I}_{0}}\right)$ is obtained as a function of energy by scanning the monochromator over the specified energy range.

To take care of the EXAFS oscillations in the absorption spectra, the energy-dependent absorption coefficient *μ*(*E*) has been converted to absorption function *χ*(*E*) defined as follows:

1 $${{{{{{\rm{\chi }}}}}}}^{(E)}=\frac{\mu \left(E\right)-{\mu }_{0}(E)}{\triangle {\mu }_{0}({E}_{0})}$$

where absorption edge energy is the bare atom background and is the step in the value at the absorption edge. After converting the energy scale to the photoelectron wave number scale (*k*) as defined by

2 $$k=\sqrt{\tfrac{2m(E-{E}_{0})}{{{{\hbar }}}^{2}}}$$

The energy-dependent absorption coefficient has been converted to the wave number-dependent absorption coefficient *χ*^(*k*)^, where is the electron mass. Finally, *χ*^(*k*)^ is weighted by *k*_2_ to amplify the oscillation at high, and the functions *χ*^(*k*)^ *k*_2_ are Fourier transformed in space to generate the *χ*^(*r*)^ vs. *r* (or FT-EXAFS) spectra in terms of the real distances from the center of the absorbing atom. The range used for the Fourier transform is 2–10 Å^−1^. It should be mentioned here that a set of EXAFS data analysis programs available within the Demeter software package^29^ have been used for the reduction and fitting of the experimental EXAFS data. This includes data reduction and Fourier transformation to derive the versus plots from the absorption spectra, generation of the theoretical EXAFS spectra starting from an assumed crystallographic structure and finally fitting of the experimental versus data with the theoretical ones using the FEFF 6.0 code.

#### Photocatalytic hydrogen generation activity

The photocatalytic light-driven hydrogen generation activity experiment has been carried out using the previously followed methods. In particular, a 100 mL double-jacketed Pyrex reactor containing 10 mg of the photocatalyst was dispersed in a mixture containing 2 mL of TEOA and 18 mL of deionized water. The solution was initially subjected to evacuation to abolish the dissolved gases followed by nitrogen purging to maintain an inert atmosphere. The photoreactor was then placed in front of the 300 W Xe lamp for light irradiation, the gas so evolved during the reaction has been analyzed using Perkin Elmer Clarus 590 GC equipped with TCD using Nitrogen as the carrier gas.

The turnover number (TON) of the hydrogen evolution rate can be estimated according to the given equation

$${{{{{\rm{TON}}}}}}=\frac{2\times {{{{{\rm{amount}}}}}}\,{{{{{\rm{of}}}}}}\,{{{{{\rm{produced}}}}}}\,{{{{{\rm{hydrogen}}}}}}}{{{{{{\rm{amount}}}}}}\,{{{{{\rm{of}}}}}}\,{{{{{\rm{dye}}}}}}\,{{{{{\rm{catalyst}}}}}}\,{{{{{\rm{used}}}}}}}$$

#### Apparent quantum yield (AQY) calculation

The apparent quantum yield (AQY) was calculated based on the experimental conditions used in this work. We have used an optical power or energy meter (Newport, Model: 842-PE) to determine the number of incident photons (*N*_photons_). The values of *N*_photons_ and AQY (%) were calculated using the following equations^43,44^:

$${{N}}_{{{{{{\rm{photon}}}}}}}=\frac{{P}{\lambda }{t}}{{hc}}$$

Here, *P* represents the power of the light (1.92 J s^−1^ cm^−2^) in an area of 12.62 cm^2^, *λ* is the wavelength of the light (420 nm), *t* is the duration of irradiation (3 h), *h* is Planck’s constant (6.626 × 10^−34^ J s) and *c* is the velocity of light (3 × 10^8^ m s^−1^).

$${{{{{\rm{AQY}}}}}}=\frac{2\times {{{{{\rm{numbers}}}}}}\,{{{{{\rm{of}}}}}}\,{{{{{\rm{evolved}}}}}}\,{{{{{{\rm{H}}}}}}}_{2}\,{{{{{\rm{moecule}}}}}}}{{{{{{\rm{numbers}}}}}}\,{{{{{\rm{of}}}}}}\,{{{{{\rm{incident}}}}}}\,{{{{{\rm{photons}}}}}}\,({{N}}_{{{{{{\rm{photon}}}}}}})}\times 100$$

#### Photoelectrochemical studies

The photoelectrochemical studies of the photocatalyst have been carried out using a three-electrode potentiostat (CH Instrument, CHI 6005E) consisting of Ag/AgCl as the reference electrode, platinum wire as the counter electrode, and photocatalyst-coated ITO film acting as the working electrode in 0.1 M Tetrabutylammonium perchlorate (Bu_4_NClO_4_) electrolytic solution.

## Supplementary information

Supplemental Material

## Data Availability

All the data supporting the findings of this study are available in the article and its supplementary information.
