# Supplementary material for: Anisotropic phenanthroline-based ruthenium polymers grafted on a titanium metal-organic framework for efficient photocatalytic hydrogen evolution
Source: Commun Chem. 2022 Dec 3;5:165. doi: 10.1038/s42004-022-00763-8 (PMC9814133; doi:10.1038/s42004-022-00763-8)
Supplement: Supplementary file 1 — Supplemental Material [file 42004_2022_763_MOESM1_ESM.pdf]

Supporting Information

Anisotropic phenanthroline-based ruthenium polymers grafted on a titanium metal-organic framework for efficient photocatalytic hydrogen evolution

Spandana Gonuguntla,[1,6] Saddam Sk, [1,6] Anjana Tripathi,[2] Ranjit Thapa,[2] Gopinath Jonnalagadda,[3,6] Chandrani Nayak,[4] Dibyendu Bhattacharyya,[4] S. N. Jha,[5] Annadanam V. Sesha Sainath,[3,6] Vijayanand Perupogu,[1,6] Ujjwal Pal [1,6*]

[1]Department of Energy & Environmental Engineering, CSIR-Indian Institute of Chemical Technology, Hyderabad-500007, India. E-mail: upal03@gmail.com; ujjwalpal@iict.res.in
[2]Department of Physics, SRM university-AP, Amravati-522502, Andhra Pradesh, India
[3]Polymer and Functional Materials, Fluoro and Agrochemicals Department, CSIR-Indian Institute of Chemical Technology, Hyderabad-500007, India.
[4]Atomic and Molecular Physics Division, Bhabha Atomic Research Centre, Mumbai-400085, India.
[5]Beamline Development and Application Section, Bhabha Atomic Research Centre, Mumbai-400085, India
[6]Academy of Scientific and Innovative Research (AcSIR), Ghaziabad-201002, India.

Characterizations. The phase identification and chemical modifications of the compounds have been characterized through the X-ray diffractometer (D8 Advance Bruker with CuKα) set with a scanning range of 2θ = 2° to 70°, Fourier Transformation-Infrared (FTIR) recorded at a scanning range of 4000-400 cm^{-1} over a resolution of 2 cm^{-1}. Morphological studies and elemental distribution of the prepared composites have been examined through the Field Emission Scanning Electron Microscope (FESEM) equipped with an Oxford energy-dispersive analysis of X-ray (EDAX) detector. Furthermore, high-resolution transmission electron microscopy (HRTEM) of model JEOL 2100 LaB6 operating at 200 kV where the sample was dispersed in ethanol. UV-vis Diffuse Reflectance Spectroscopy (DRS) (Perkin – Elmer Lambda 750), where we used Barium Sulphate (Ba_2SO_4) as reference material. Photoluminescence and Time-Correlated Single Photon Count (TCSPC) studies were carried out through the FlouroLog3-Triple Illuminator, IBH Horiba Jobin Yvon instrumental setup. An atomic force microscope (AFM, Veeco, Santa Barbara, CA, USA) with a scan rate of 0.8 Hz in contact mode was used to record the images and surface roughness of the films. AFM samples were prepared by simple drop-casting method on Si (100) surface substrate. A homogeneous solution of P4T & P4T-D samples in ethanol is drop-casted onto Si-surface and dried at 80 ̊C. Surface and particle analysis have been carried out through the Brunauer-Emmett-Teller (BET) (Quantachrome Instrument, USA; Model No. NOVA 1000e) through

the Nitrogen adsorption/desorption studies at 77 K using a surface area analyzer, Chemical bonding changes have been analyzed through the X-ray Photoelectron Spectroscopy (XPS) using XPS, Kratos-Axis Supra 165 with Mg Kα irradiation and a pressure of 10^{-9} Torr. Positron annihilation lifetime spectra were measured with a lifetime spectrometer constructed from two identical BaF2 scintillation detectors via fast-fast coincidence. The resolution of the spectrometer was 175 ps. The spectra were acquired by immersing a 10 micro-Curie Na-22 source encapsulated between two identical 8-micron polyimide films in the powder samples. The spectra were acquired under ambient conditions. The spectra were analyzed using PALSfit software using appropriate corrections for the positrons annihilating in the source and encapsulating polyimide films[1]. Photocatalytic hydrogen evolution reaction (HER) has been analyzed using Perkin Elmer Clarus 590 GC equipped with 5Å molecular sieves column under nitrogen flow under 450 W Xe lamp light irradiation (Newport, USA) and the consecutive photoelectrochemical measurements were examined through the three-electrode potentiostat system (CH Instrument, CHI 6005E) where Ag/AgCl as reference electrode, platinum wire as the counter electrode and photocatalyst coated ITO film acts as working electrode in 0.1 M Tetrabutylammonium perchlorate (Bu_4NClO_4) as an electrolytic solution.

NMR analysis. As reported earlier[2], 10-(1,10-phenanthroline-3-acyloxy)decylacrylatebis(1,10-phenanthroline)ruthenium(II) hexaflouro phosphate (**AR**) monomer was synthesized by 1,10-phenanthroline having long alkyl chain acrylate reaction with $Ru(Phen)_2Cl_2$ and purified by column chromatography using acetonitrile as eluent. Further, its well-defined macromolecular architectures (**P3** and **P4**) were synthesized by the atom transfer radical polymerization method as reported earlier[3].

AR. ESI–MS: m/z: 1014 $[PDAR]^+$. IR (KBr, cm^{-1}, *v*): 3025 (C-H, Ar), 2928 and 2855 (C-H), 1715 (ester C=O), 1602 (C=C, acrylic), 1505 (C=C, Ar), 1237 and 1196 (–C–O–C–) and 558 (Ru-*N*). ^{1}H-NMR (300 MHz, $CDCl_3$, δ): 8.66 (m, 4H), 8.5 (d, *J* = 8.3, 1H), 8.3 (d, *J* = 6.6, 4H), 8.2 (s, 2H), 8.0 (m, 4H), 7.98 (d, *J* = 4.72, 1H), 7.92 (d, *J* = 5.0, 1H), 7.8-7.7 (m, 4H), 7.6 (m, 1H), 7.57 (d, *J* = 2.0, 1H), 6.3 (dd, *J* = 17.3, J = 1.5, 1H), 6.15 (dd, *J* = 17.7, *J* =10.3, 1H), 5.8 (dd, *J* = 10.3 and 1.32 1H), 4.11 (t, *J* = 6.6, 4H), 1.7 (p, *J* = 6.7, *J* = 13.6 2H), 1.64 (p, *J* = 6.7, *J* = 13.6 2H) and 1.27 ppm (m, 12H). ^{13}C-NMR (75 MHz, $CDCl_3$+DMSO-d_6, δ): 171.08, 161.48, 157.30, 152.30, 149.43, 145.95, 142.10, 137.07, 135.48, 133.49, 131.35, 129.98, 121.16, 107.97, 74.53, 69.30, 33.91, 33.63 and 30.42 ppm.

PPDAR-3. M_n from NMR:15,550 g/mol. IR (KBr, cm^{-1}, *ν*): 3025 (C-H, Ar), 2928 and 2855 (C-H), 1724 (ester C=O), 1237 and 1196 (–C–O–C–) and 581(Ru–*N*). ^{1}H-NMR (400 MHz, DMSO-d_6, *δ*): 8.76, 8.71, 8.40, 8.21, 8.12, 7.98, 7.77, 7.67, 4.29, 4.08, 1.60, 1.48, 1.15 and 0.85 ppm. ^{13}C-NMR (75 MHz, DMSO-d_6, *δ*): 172.19, 155.84, 152.89, 147.29, 144.94, 141.40, 137.11, 131.47, 130.40, 128.03, 126.47, 125.13, 69.15, 64.04, 54.84, 28.53 and 25.28 ppm.

PPDAR-4. M_n from NMR: 12,730 g/mol. IR (KBr, cm^{-1}, *ν*): 3025 (C-H, Ar), 2928 and 2855 (C-H), 1724 (ester C=O), 1237 and 1196 (–C–O–C–) and 581(Ru–*N*). ^{1}H-NMR (300 MHz, DMSO-d_6, *δ*): 8.77, 8.41, 8.23, 8.10, 8.02, 7.79, 7.69, 4.05, 1.78, 1.56, 1.24 and 1.09 ppm. ^{13}C-NMR (75 MHz, DMSO-d_6, *δ*): 175.49, 161.27, 157.53, 152.69, 146.61, 142.04, 135.65, 133.28, 131.54, 70.13, 59.18, 34.15, 30.77 and 20.65 ppm.

Atomic Force Microscopy (AFM) analysis

AFM samples were prepared by simple drop-casting method on Si (100) surface substrate. A homogeneous solution of P4T & P4T-D samples in ethanol is drop-casted onto Si-surface and dried at 80 °C.

Computational Details

All DFT calculations were carried out with the Vienna Ab-initio Simulation Package (VASP)[4]. The Perdew-Burke-Ernzerhof (PBE) within Generalized Gradient Approximation (GGA)[5] functional was adopted to treat the exchange-correlation interactions. Configurations were allowed to relax until an energy difference of 10−4 eV and the maximum force was converged to lower than 0.01 eV/Å with the kinetic energy cut-off of 450 eV. The thickness of the vacuum layer was set to be 30 Å to sufficiently avoid the interactions between mirror images. The Γ point was sampled due to the size of the system for geometry optimization.

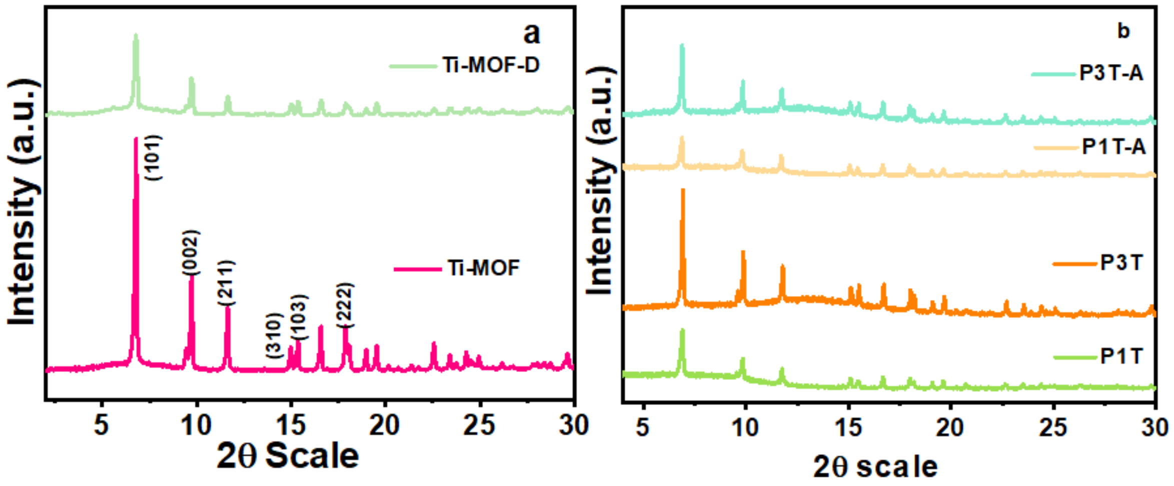

Supplementary Figure 1: XRD analysis of **a** Ti-MOF, Ti-MOF-D, **b** P1T, P3T, P1T-A, P3T-A.

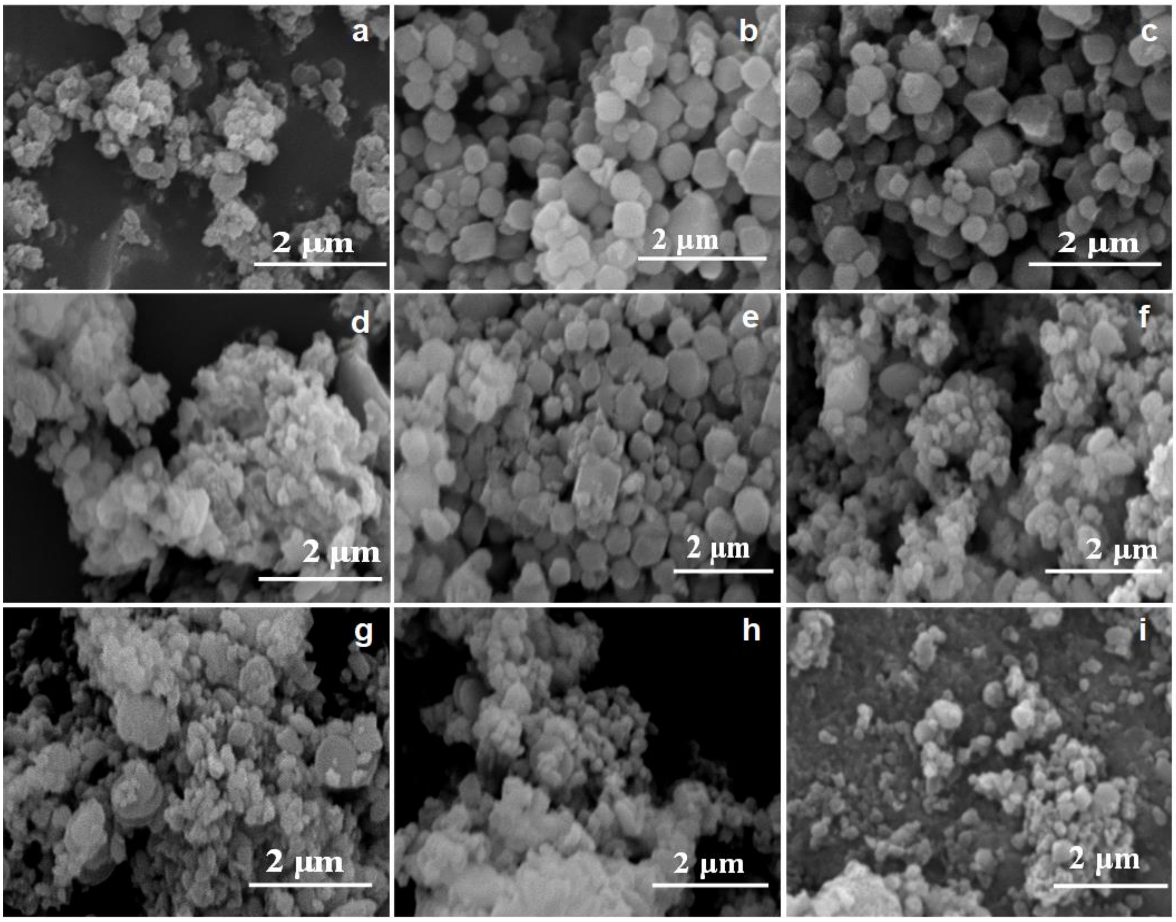

Supplementary Figure 2: SEM analysis of **a** P1T, **b** P3T, **c** P4T, **d** P1T-A, **e** P3T-A, **f** P4T-A, **g** P1T-D, **h** P3T-D, and **i** P4T-D respectively.

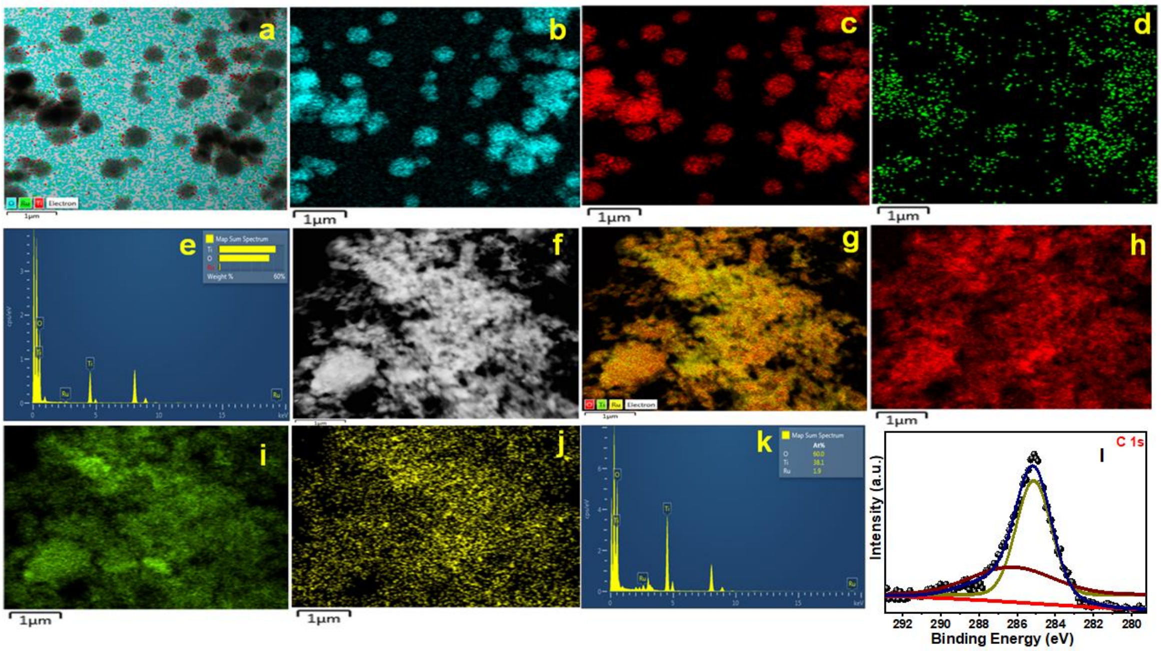

Supplementary Figure 3: a HAADF image of P4T, **b-e** Elemental mapping of O, Ti, Ru, **f** HAADF image of P4T-D, **g-k** Elemental mapping of O, Ti, Ru elements, **l** XPS spectra of P4T C 1s.

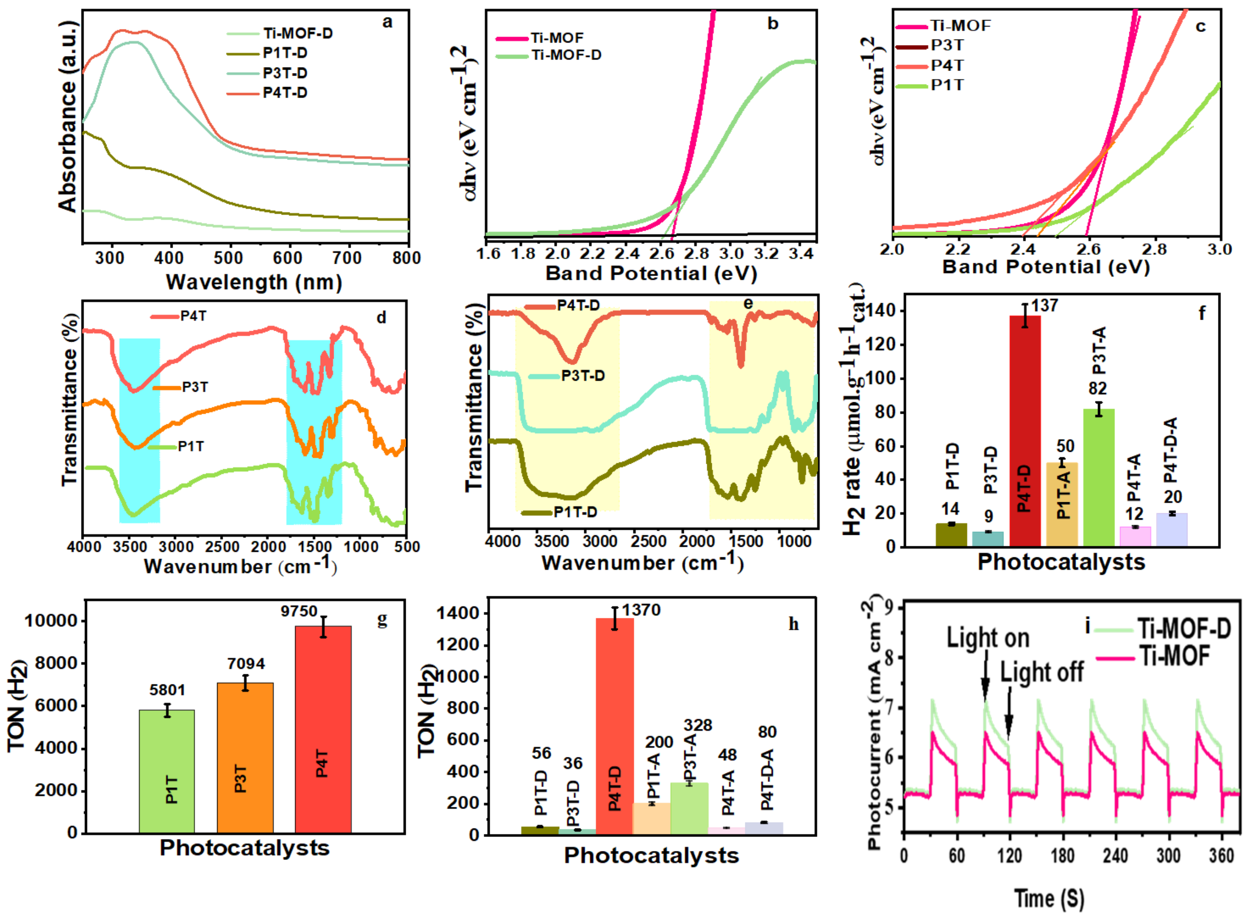

Supplementary Figure 4: a UV-vis absorbance spectra of Ti-MOF-D, P1T-D, P3T-D, P4T-

D, bandgap potentials of **b** Ti-MOF, Ti-MOF(D), **c** P1T, P3T, P4T, FTIR analysis of **d** P1T, P3T, P4T, **e** P1T-D, P3T-D, P4T-D, and **f** Histogram of H_2 evolution rate under visible light of P1T-D, P3T-D, P4T-D, P1T-A, P3T-A, P4T-A, Error bar diagram of **g** P1T, P3T, P4T, **h** P1T-D, P3T-D, P4T-D, P1T-A, P3T-A, P4T-A, P4T-D-A, and **i** transient photocurrent stability of Ti-MOF, and Ti-MOF-D, respectively.

Atomic Force Microscopy (AFM) analysis

AFM samples were prepared by simple drop-casting method on Si (100) surface substrate. A homogeneous solution of P4T & P4T-D samples in ethanol is drop-casted onto Si-surface and dried at 80 °C.

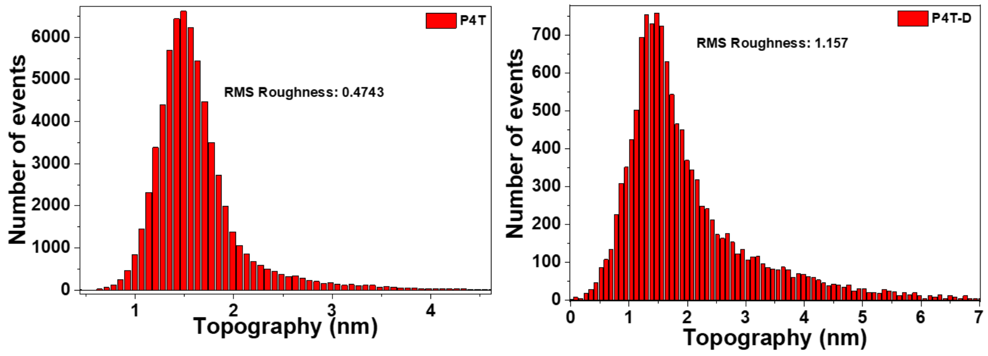

Supplementary Figure 5: RMS surface roughness topography of P4T and P4T-D.

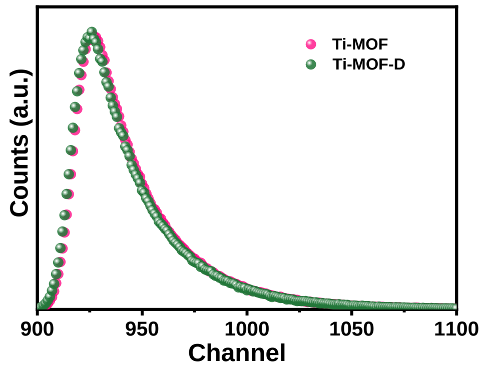

Supplementary Figure 6: Positron lifetime spectra of Ti-MOF and Ti-MOF-D.

Table S1: Positron lifetime parameters of Ti-MOF and Ti-MOF-D samples.

Sample	τ_1 (ps)	I_1 (%)	τ_2 (ps)	I_2 (%)
Ti-MOF	229	34	373	66
Ti-MOF-D	241	42	389	58

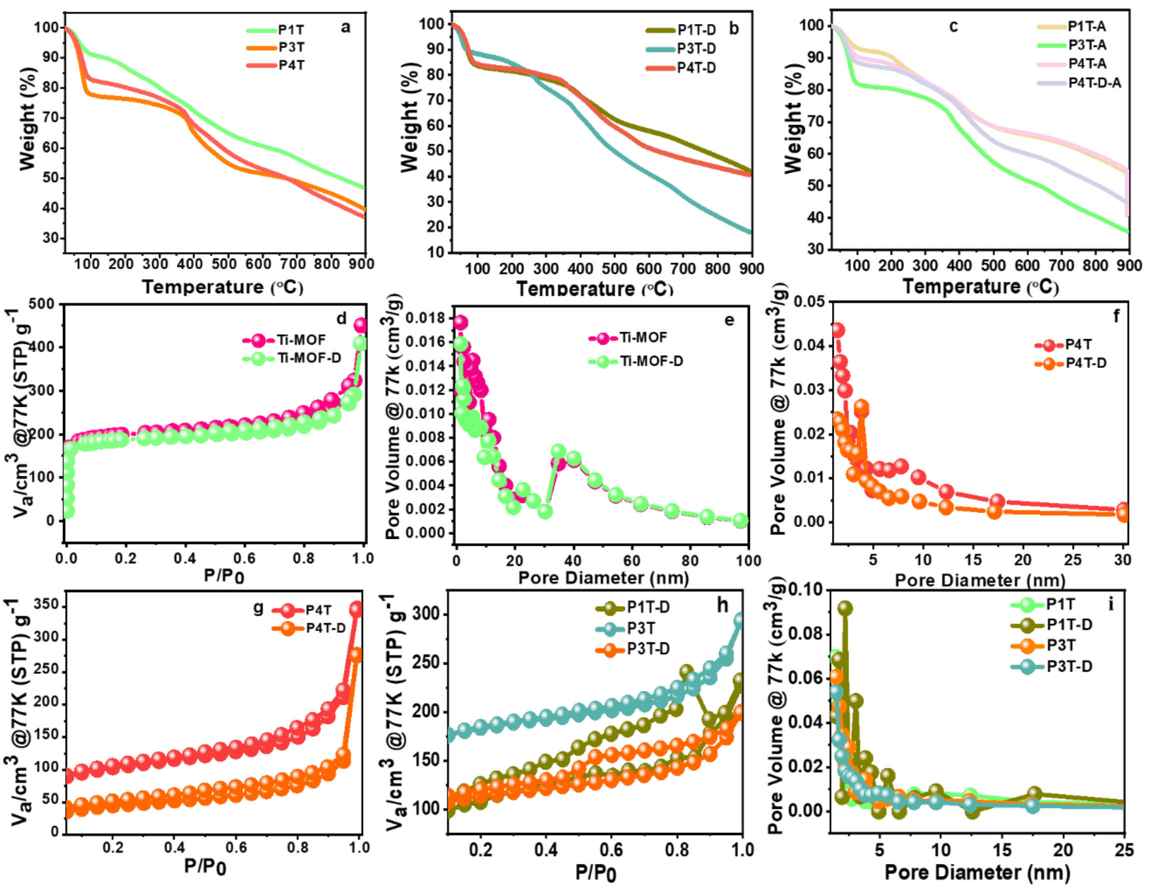

Supplementary Figure 7: Thermogravimetric analysis of **a** P1T, P3T, P4T, **b** P1T-D, P3T-D, P4T-D, and **c** P1T-A, P3T-A, P4T-A, and P4T-D-A, **d** N_2 adsorption-desorption isotherm of Ti-MOF and Ti-MOF-D, pore size distribution curves of **e** Ti-MOF and Ti-MOF-D, and **f** P4T, P4T-D, **g** P4T, P4T-D, **h** P1T-D, P3T, P3T-D, and **i** P1T, P1T-D, P3T, P3T-D respectively.

Table S2: Comparative surface area and pore distribution analysis of photo-catalyst.

S. No	Catalyst	Surface Area [m^2 g^{-1}]	Mean Pore Diameter [nm]	Total Pore Volume [cm^3 g^{-1}]
1	Ti-MOF	756.04	3.5404	0.6692
2	Ti-MOF-D	713.88	3.5156	0.6274
3	P4T	199.05	1.422	0.454
4	P4T-D	133.45	3.829	0.402

Table S3: Average lifetime measurements of the composites

S. No	Photocatalyst	τ1 (ns)	τ2 (ns)	τ3 (ns)	Average lifetime (ns)
1	P1T	0.15	0.42	11.48	0.16
2	P3T	0.05	0.46	11.255	0.061
3	P4T	0.25	0.55	5.918	0.31

Table S4: Comparative study of previous results:

S. No	Catalyst	light irradiation & SED	Hydrogen production	TON	AQY (%)	Ref.
1	NH_2-MIL-125/TiO_2/CdS	300 W Xe lamp, Na_2S and Na_2SO_3	2997 μmol g^{-1} h^{-1}	-	4.81	6
2	ML200	300 W Xe lamp, TEOA	127 mmol g^{-1} h^{-1}	-	-	7
3	PCP4e	150 W Xe lamp, TEA	33 μmol h^{-1}	-	0.34	8
4	Ti-MOF-Ru(tpy)$_2$	500 W Xe lamp, TEOA	11 μmol	-	-	9
5	P64	AM1.5G, TEOA	6039 μmol g^{-1} h^{-1}	-	20.7	10
6	Cu_2O/NH_2-MIL-125(Ti) MOF	300 W SS-EM, TEOA	488 μmol g^{-1} h^{-1}	-	1.4	11
7	Ru-MIL-125-NH_2	300 W Xe lamp, TEOA	426 μmol g^{-1} h^{-1}	-	-	12
8	NH_2-ML-125/Co(dmgH)$_2$ (3 wt%)	300 W Xe lamp, TEOA	2195 μmol g^{-1} h^{-1}	-	-	13
9	[Co^{II}(TPA)Cl][Cl]-MIL-125-NH_2	300 W Xe lamp, TEOA	553 μmol g^{-1} h^{-1}	-	-	14
10	Ti-MOF	300 W Xe lamp, TEOA	238 μmol g^{-1} h^{-1}	-	0.52	This work
11	Ti-MOF-D	300 W Xe lamp, TEOA	349 μmol g^{-1} h^{-1}	-	0.76	This work
12	P1T	300 W Xe lamp, TEOA	1450 μmol g^{-1} h^{-1}	5801	3.17	This Work
13	P3T	300 W Xe lamp, TEOA	1774 μmol g^{-1} h^{-1}	7094	3.88	This Work

**14**	**P4T**	**300 W Xe Lamp, TEOA**	**2438 μmol g^{-1} h^{-1}**	**9750**	**5.33**	**This Work**
15	P1T-D	300 W Xe lamp, TEOA	14 μmol g^{-1} h^{-1}	56	0.03	This Work
16	P3T-D	300 W Xe lamp, TEOA	9 μmol g^{-1} h^{-1}	36	0.01	This Work
17	P4T-D	300 W Xe lamp, TEOA	137 μmol g^{-1} h^{-1}	1370	0.30	This Work
18	P1T-A	300 W Xe lamp, TEOA	50 μmol g^{-1} h^{-1}	200	0.10	This Work
19	P3T-A	300 W Xe lamp, TEOA	82 μmol g^{-1} h^{-1}	328	0.17	This Work
20	P4T-A	300 W Xe lamp, TEOA	12 μmol g^{-1} h^{-1}	48	0.02	This Work
21	P4T-D-A	300 W Xe lamp, TEOA	20 μmol g^{-1} h^{-1}	80	0.04	This Work

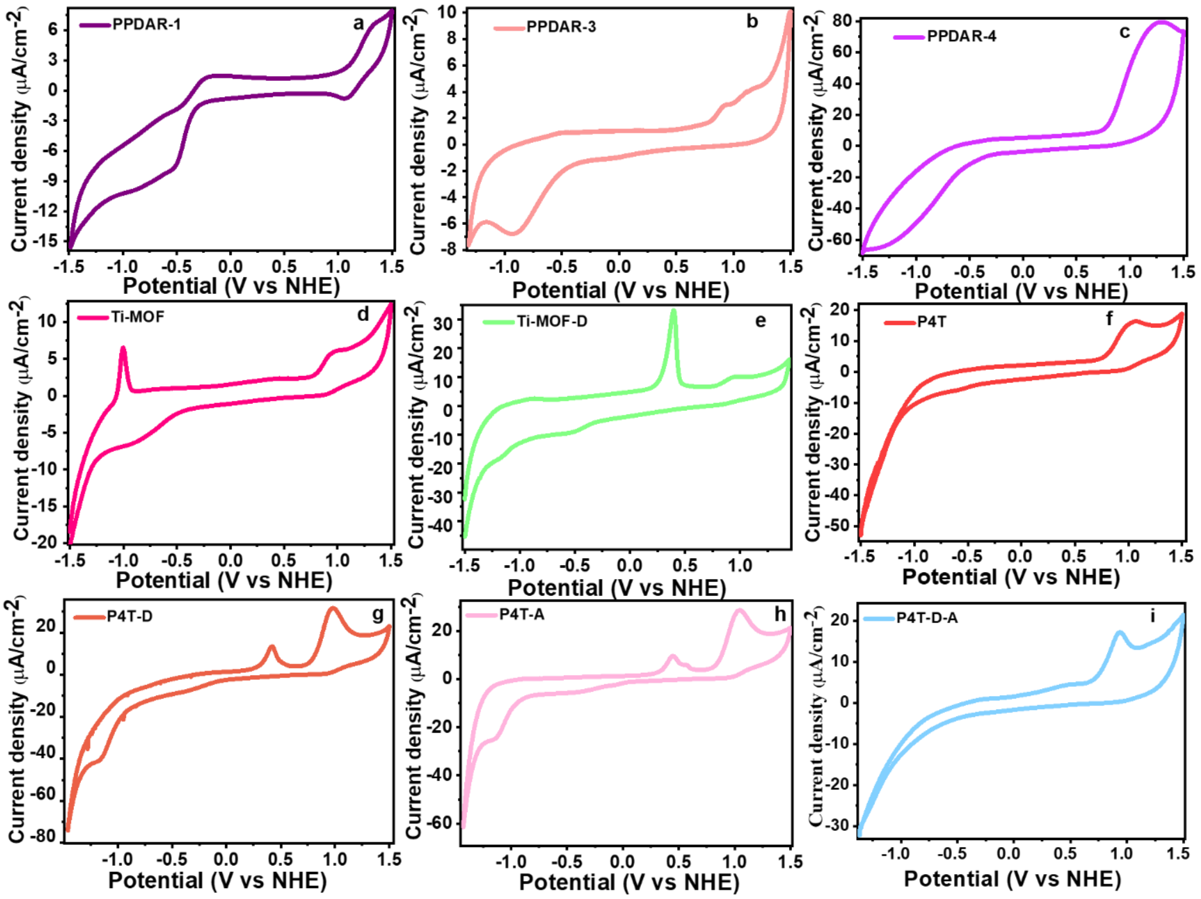

Supplementary Figure 8: Cyclic Voltammetry studies of **a** PPDAR-1, **b** PPDAR-3, **c** PPDAR-4, **d** Ti-MOF, **e** Ti-MOF-D, **f** P4T, **g** P4T-D, **h** P4T-A, and **i** P4T-D-A respectively.

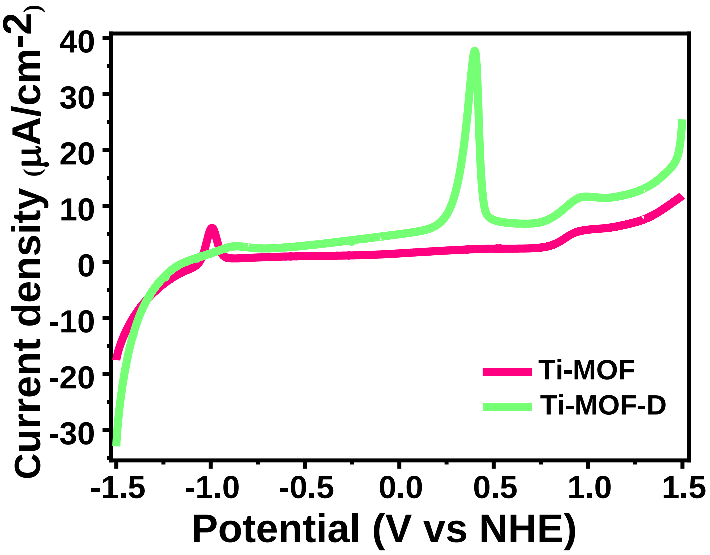

Supplementary Figure 9: Linear Sweep Voltammetry studies of Ti-MOF and Ti-MOF-D.

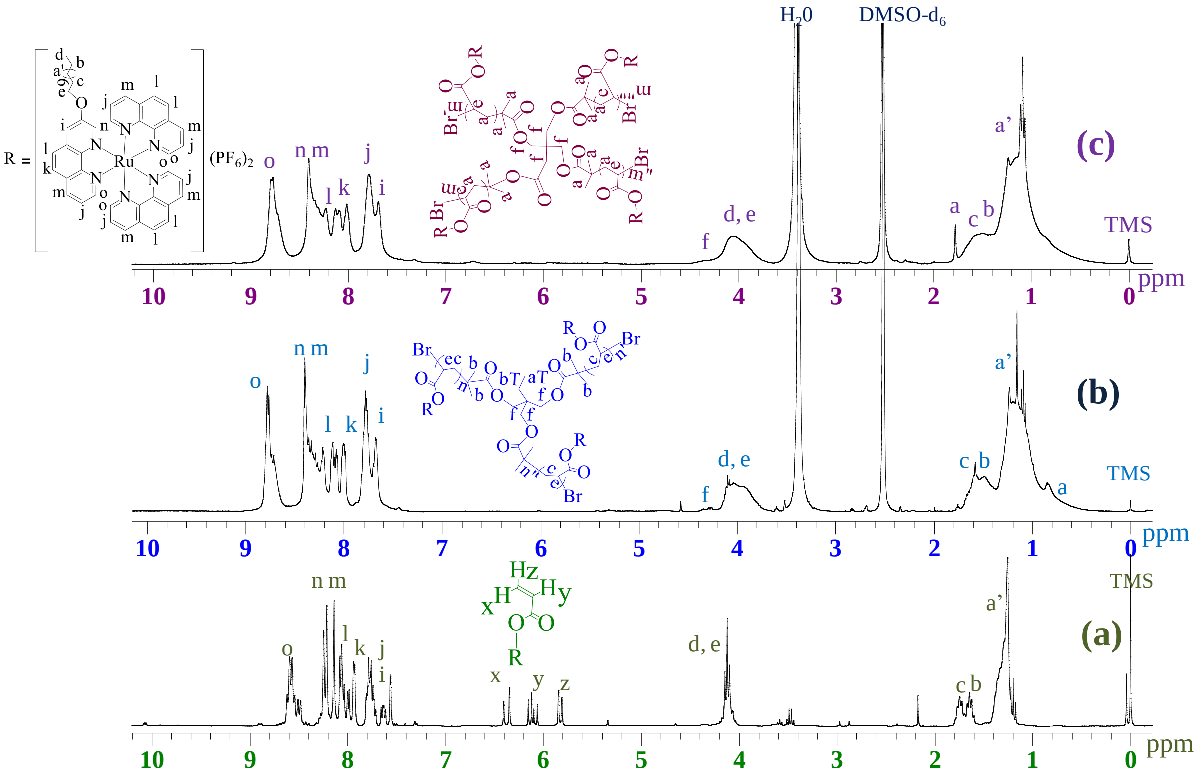

Supplementary Figure 10: ^{1}H NMR spectra of **a** AR in $CDCl_3$, and **b** PPDAR-3 and **c** PPDAR-4 in DMSO-d_6.

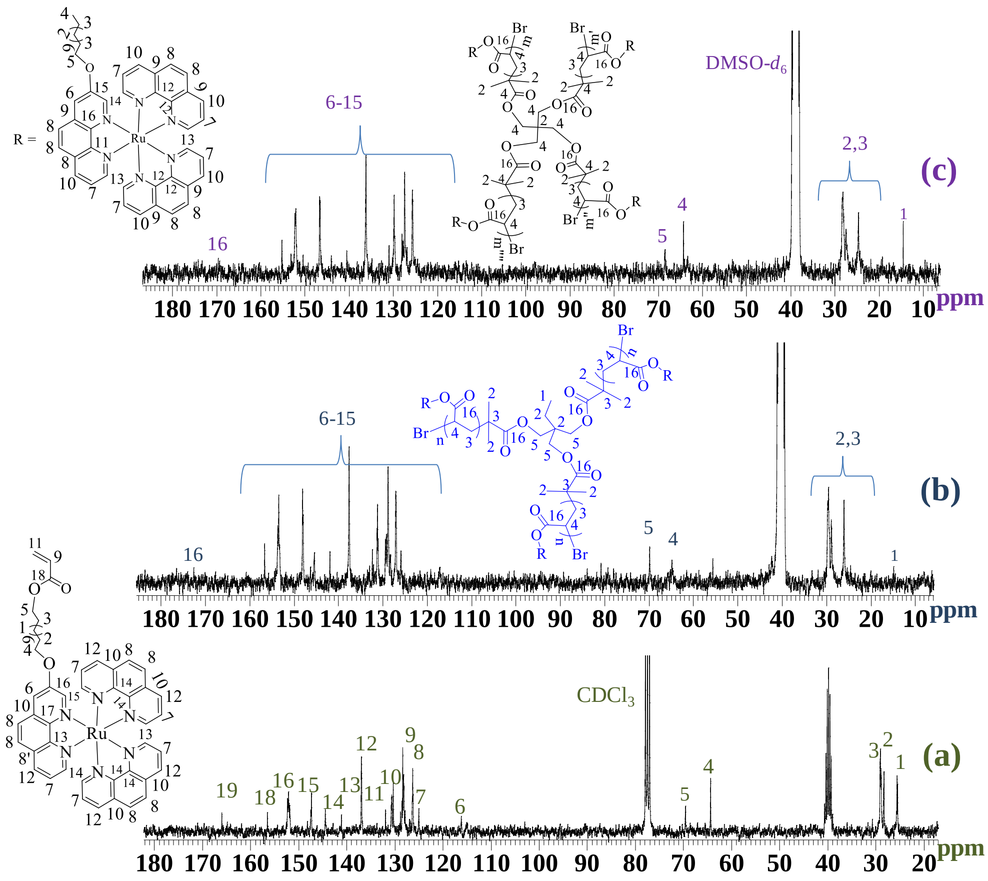

Supplementary Figure 11: ^{13}C-NMR spectra of **a** AR in $CDCl_3$, and **b** PPDAR-3 and **c** PPDAR-4 in DMSO-d_6.

References:

1. Olsen, J.V., Kirkegaard, P., Pedersen, N.J., Eldrup, M., PALSfit: a new program for the evaluation of positron lifetime spectra, *Physica status solidi C*, **4,** 4004-4006 (2007).
2. Karthik, P., Vinoth, R., Zhang, P., Choi, W., Balaraman, E., Neppolian, B. – Interaction between metal-organic framework and reduced graphene oxide for visible-light photocatalytic H_2 production. *ACS Appl. Energy Mater.* **1**, 1913-1923 (2018).
3. Gopinath, J., Park, K. H., Kim, S., Santosh, V., Sainath, A. V. S., Dhayal, M., Phenanothroline-based ruthenium complexes for enhanced charge transportation in solvent-free ionic liquid electrolyte. *J. Mater. Sci.* **52**, 10545-10556 (2017).
4. Kresse, G., Furthmuller, J. Efficiency of ab-inito total energy calculations for metals and semiconductors using a plane-wave basis set. *Comput. Mater. Sci.* **6**, 15–50 (1996**)**.

5. Perdew, J. P., Burke, K., Ernzerhof, M. Generalized Gradient Approximation Made Simple. *Phys. Rev. Lett.* **77**, 3865–3868 (1996).
6. Bibi, R., Huang, H., Kalulu, M., Shen, Q., Wei, L., Oderinde, O., Li, N., Zhou, J. Synthesis of Amino-Functionalized Ti-MOF Derived Yolk–Shell and Hollow Heterostructures for Enhanced Photocatalytic Hydrogen Production under Visible Light. *ACS Sustainable Chem. Eng.* **7**, 4868-4877 (2019).
7. Sohail, M., Kim, H., Kim, T. W. Enhanced photocatalytic performance of a Ti-based metal-organic framework for hydrogen production: Hybridization with ZnCr-LDH nanosheets. *Scientific Reports.* **9**, 7584 (2019).
8. Li, L., Cai, Z., Wu, Q., Lo, W., Zhang, N., Chen, L.X., Yu, L. Rational design of porous conjugated polymers and roles of residual palladium for photocatalytic hydrogen production. *J. Am. Chem. Soc.* **138**, 7681–7686 (2016).
9. Toyao, T., Saito, M., Dohshi, S., Mochizuki, K., Iwata, M., Higashimura, H., Horiuchi, Y., Matsuoka, M. Development of a Ru complex-incorporated MOF photocatalyst for hydrogen production under visible-light irradiation. *Chem. Commun.,* **50,** 6779-6781 (2014).
10. Bai, Y., Wilbraham, L., Slater, B. J., Zwijnenburg, M. A., Sprick, R. S., Cooper, A. I. Accelerated discovery of organic polymer photocatalysts for hydrogen evolution from water through the integration of experiment and theory. *J. Am. Chem. Soc.* **141**, 9063−9071 (2019).
11. Karthik, P., Balaraman, E., Neppolian, B. Efficient solar light-driven H_2 production: post-synthetic encapsulation of a Cu_2O co-catalyst in a metal-organic framework (MOF) for boosting the effective charge carrier separation. *Catal. Sci. Technol.* **8**, 3286-3294 (2018).
12. Zhang, F., Zhang, B., Feng, J., Tan, X., Liu, L., Liu, L., Han, B., Zheng, L., Zhang, J., Tai, J., Zhang, J. Highly mesoporous Ru-MIL-125-NH_2 produced by supercritical fluid for efficient photocatalytic hydrogen production. *ACS Appl. Energy Mater.* **2**, 4964-4970 (2019).
13. Luo, S, Liu, X., Wei, X., Fu, W., Lu, P., Li, X., Jia, Y., Ren, Q., He, Y. Noble-metal-free cobaloxime coupled with metal-organic frameworks NH_2-MIL-125: A novel bifunctional photocatalyst for photocatalytic NO removal and H_2 evolution under visible light irradiation. *J. Hazar. Mater.,* **399**, 122824 (2020).

14. Li, Z., Xiao, J-D., Jiang, H-L. Encapsulating a Co(II) Molecular Photocatalyst in Metal-Organic Framework for Visible-Light-Driven H_2 Production: Boosting Catalytic Efficiency via Spatial Charge Separation. *ACS Catal.* **6**, 5359-5365 (2016).
